# Rhythmic oscillations of the microRNA miR-96-5p play a neuroprotective role by indirectly regulating glutathione levels

**DOI:** 10.1038/ncomms4823

**Published:** 2014-05-07

**Authors:** Chisato Kinoshita, Koji Aoyama, Nobuko Matsumura, Kazue Kikuchi-Utsumi, Masahiko Watabe, Toshio Nakaki

**Affiliations:** 1 Department of Pharmacology, Teikyo University School of Medicine, 2-11-1 Kaga, Itabashi-ku, Tokyo 173-8605, Japan; 2 General Medical Education Center (G-MEC), Teikyo University School of Medicine, 2-11-1 Kaga, Itabashi-ku, Tokyo 173-8605, Japan

## Abstract

Glutathione (GSH) is a key antioxidant that plays an important neuroprotective role in the brain. Decreased GSH levels are associated with neurodegenerative diseases such as Parkinson’s disease and Alzheimer’s disease. Here we show that a diurnal fluctuation of GSH levels is correlated with neuroprotective activity against oxidative stress in dopaminergic cells. In addition, we found that the cysteine transporter excitatory amino acid carrier 1 (EAAC1), which is involved in neuronal GSH synthesis, is negatively regulated by the microRNA miR-96-5p, which exhibits a diurnal rhythm. Blocking miR-96-5p by intracerebroventricular administration of an inhibitor increased the level of EAAC1 as well as that of GSH and had a neuroprotective effect against oxidative stress in the mouse substantia nigra. Our results suggest that the diurnal rhythm of miR-96-5p may play a role in neuroprotection by regulating neuronal GSH levels via EAAC1.

## Abstract (web summary)

Glutathione is a key antioxidant that plays an important neuroprotective role in the brain. Here, Kinoshita *et al.* show that levels of glutathione exhibit diurnal fluctuations that are indirectly regulated by the microRNA miR-96-5p, and that this microRNA plays a neuroprotective role against oxidative stress.

## Graphical abstract

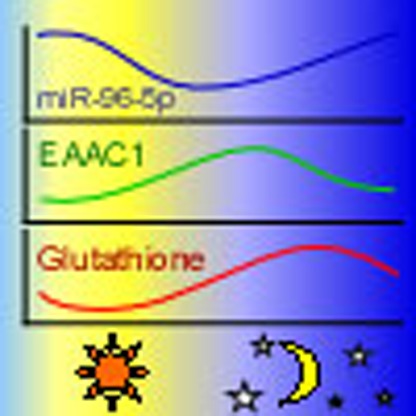

The balance between oxidants and antioxidants is a key factor for normal brain function^1^. The imbalance of redox states caused by an excess of oxidants and/or a depletion of antioxidants is defined as an oxidative-stress state^2^. Glutathione is an especially important antioxidant in the central nervous system because of the lower activity of major antioxidant enzymes such as superoxide dismutase and catalase in the brain^3^. Glutathione exists in both a reduced form (GSH) and an oxidized form (GSSG), functioning in various redox reactions. Depletion of GSH in the brain is a known cause of neurodegenerative diseases such as Parkinson’s disease (PD). PD is characterized by a selective loss of dopaminergic neurons in the substantia nigra pars compacta (SNc)^4^.

GSH is a tripeptide composed of cysteine, glutamate and glycine^5^. Among these amino acids, cysteine is the rate-limiting factor, since the concentrations of glutamate and glycine in neurons are sufficient. Although cystine is generally known as a source of cysteine, neurons do not express the cystine transport system in mature brains, and thus cysteine is considered a major determinant for intracellular GSH synthesis in neurons.

One of the important factors regulating GSH synthesis is excitatory amino acid carrier 1 (EAAC1), a member of the sodium-dependent excitatory amino acid transporter (EAAT) family. Unlike other EAATs, EAAC1 is selectively enriched in the neurons of the central nervous system^6^. It was indicated that the transport of cysteine, rather than that of glutamate, is the major function of EAAC1 (refs 7, 8). In fact, EAAC1 deficiency decreased the neuronal GSH content and increased markers of neuronal oxidative stress in the mouse brain^9^.

The circadian clock is an internal timekeeping system that allows organisms to adapt physiological and behavioural processes to environmental light/dark cycles^10^. Almost all organisms harbour this system, indicating that the circadian clock developed early in the evolution of life. In mammals, the master clock is located in the suprachiasmatic nucleus (SCN). The SCN drives endogenous rhythms and control circadian rhythms in peripheral tissues, including other brain areas such as the SNc^11^. The circadian system is regulated by several clock genes such as transcriptional activators (for example, CLOCK and BMAL1) and repressors (for example, PER1 and 2). It was shown that BMAL1-deficient mice exhibit increased levels of reactive oxygen species (ROS) and accelerated ageing, suggesting that the circadian clock is involved in ROS regulation^12^. It was also reported that sleep disorders and circadian disruptions are common in PD patients, and that their symptoms display diurnal fluctuations^13^. Together, these reports prompt the interesting theory that there may be a significant correlation between disruption of the circadian system and the misregulation of ROS homeostasis. The mechanism of this association has long been elusive, however.

MicroRNA (miRNA) is a class of small, non-coding molecules that are involved in the post-transcriptional regulation of target gene expression^14^. Many miRNAs are highly conserved across species. The sequence in the seed region, which is defined as two to eight nucleotides of miRNA, is the key for determining the target. It has been suggested that miRNAs play important roles in regulating protein levels that exhibit circadian rhythmicity^15^. A proteomic analysis in mouse liver revealed that up to 20% of the soluble proteins are rhythmic, whereas only 10% of the mRNA is rhythmic^16^, suggesting the possible involvement of post-transcriptional regulation such as miRNA regulation. Moreover, several reports have shown that PD-related genes are also regulated by miRNAs^17^. Taken together, these findings suggest complicated connections among circadian systems, PD-related gene expression and miRNA regulation, but such connections have not yet been studied.

Here we show that GSH levels display a diurnal rhythm that is correlated with neuroprotective activity against oxidative stress in dopaminergic cells. We observed that a rhythmic expression of EAAC1, an important regulator of GSH synthesis, is negatively regulated by an miRNA, miR-96-5p, which also exhibits a diurnal rhythm. The intracerebroventricular (i.c.v.) injection of a miR-96-5p inhibitor significantly increased the GSH level, EAAC1 expression and neuroprotection against oxidative stress in the mouse brain *in vivo*. These results suggest an miRNA-regulated mechanism for controlling the circadian oscillations of GSH and its neuroprotective effects.

## Results

### Diurnal variation of GSH levels and its neuroprotective activity

**Figure 1:**
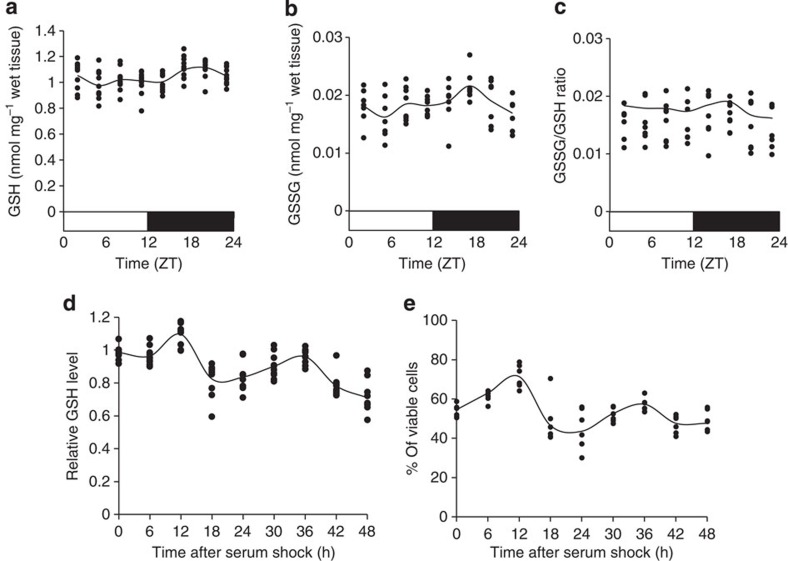
The diurnal variation of GSH level is correlated with that of protective activity against oxidative stress. (**a**) Diurnal changes in GSH levels in the mesencephalon (*n*=10, each point). The bars below the graphs indicate the light (white) and dark (black) periods. The data in the figure represent mean values and individual data points. Data were analysed by one-way ANOVA and cosinor analysis. A significant rhythmicity was detected (*P*=0.00018). (**b**) Diurnal changes in the GSSG levels in the mesencephalon (*n*=8, each point). Data were analysed by one-way ANOVA and cosinor analysis. Significant rhythmicity (*P*=0.0021) was observed. (**c**) Individual data points representing the ratio of GSSG to GSH levels in each sample (*n*=8, each point). Data were analysed by one-way ANOVA and cosinor analysis. No significant diurnal rhythm was observed (*P*=0.57). (**d**) Rhythmic changes of GSH levels in serum-shocked SH-SY5Y cells (*n*=10, each point). Data represent mean values and individual data points. Data were analysed by one-way ANOVA and cosinor analysis. A significant circadian variation (*P*=0.00023) was observed. (**e**) Time-dependent changes of viable cell percentages after H_2_O_2_ treatment for 2 h at each time point (*n*=7). Data were analysed by one-way ANOVA and cosinor analysis. Significant rhythmicity was revealed (*P*=0.0003). The number of individual data points is the same as the sample size, although some points overlap.

Although a diurnal oscillation of GSH levels in mammalian peripheral organs has been reported^18^,^19^,^20^, little has been reported on GSH rhythm in the central nervous system^21^,^22^. In the present study, to measure whether the GSH concentration exhibits a diurnal variation in the mesencephalon, which includes the SNc, we collected tissues every 3 h around the clock. Mice were fasted for 1 day before brain sampling to remove the effects of food consumption. We then observed rhythmic diurnal expression in the mesencephalon. The profile of GSH concentration displayed a diurnal rhythm with 1.2-fold changes (Cosinor; *P*=0.00018). The highest and lowest GSH levels were observed at midnight (ZT20) and at midday (ZT5), respectively (Fig. 1a).

The diurnal oscillation of the GSH concentration was also exhibited in the SCN (Cosinor; *P*=0.037), where the central oscillator is located (Supplementary Fig. 1), with the highest values detected at ZT17 and the lowest at ZT11. The diurnal rhythm of GSH levels in the brain exhibited a peak level during the night-time. A higher GSH level in the night-time and/or a lower level in the daytime may have important physiological implications.

It has also been reported that the level of GSSG displays a diurnal rhythm in the brain tissue^23^, and thus in the present study we also measured the GSSG concentration. As a result, as shown in Fig. 1b, we found rhythmic changes of the GSSG concentration in the mesencephalon (Cosinor; *P*=0.0021). The phase of its oscillation was similar to the GSH rhythm, with a peak level at ZT17 and a trough at ZT5 (Fig. 1b). The ratio of GSSG/GSH thus exhibits no diurnal rhythm (Cosinor; *P*=0.57) (Fig. 1c). These results suggest that the determinants of the rhythmic GSH concentration are more closely related to GSH synthesis than to its redox system.

To further investigate the importance of GSH rhythms, we used a serum shock protocol^24^,^25^ to synchronize the internal, self-sustained circadian clock of a neuroblastoma cell line, SH-SY5Y, which is commonly used in dopaminergic models. We performed qRT–PCR with the cells collected every 6 h after the serum shock and confirmed that exposure to a high concentration of serum shock induced the expression of a clock gene (*Per2*) and triggered a rhythmic oscillation (Cosinor; *P*=0.0028), which is comparable with other cell lines as reported^24^,^25^ (Supplementary Fig. 2b). In the serum-shocked SH-SY5Y cells, the GSH levels displayed oscillation with a period of 24-h cycles (Cosinor; *P*=0.00023) (Fig. 1d). The peak level was observed 12 h after serum shock, and the next one appeared 36 h later, which is anti-phasic to *Per2* expression, and similar to that observed *in vivo* (Fig. 1a,d and Supplementary Fig. 2a,b).

GSH is one of the most important antioxidants for protection against oxidative stress, and it is possible that its rhythmic oscillation makes neuronal protection and/or vulnerability to stress time-dependent. To test the time-dependent neuroprotection of SH-SY5Y cells, we induced oxidative stress with H_2_O_2_ every 6 h after serum shock. We determined the H_2_O_2_ concentration at 500 μM with half of the cells damaged at time =0 (Supplementary Fig. 3). The percentage of viable cells after H_2_O_2_ exposure fluctuated rhythmically over a period of 24 h (Cosinor; *P*=0.0003), which is in-phase with GSH rhythm (Fig. 1d,e).

The highest percentage was observed 12 h after serum shock; the next peak appeared 24 h later. This coincident phase between the rhythmic GSH level and viable cell percentages was also observed in non-dopaminergic HEK293 cells (Supplementary Fig. 4b,c), suggesting that the rhythmic oscillation of GSH levels, regardless of cell type, regulates the rhythmic protective activity against oxidative stress.

### Diurnal fluctuation of EAAC1 protein expression

To identify the genes that regulate GSH oscillation, we searched possible gene candidates that encode the major regulatory factor of GSH synthesis, using the NCBI Gene Expression Omnibus ( http://www.ncbi.nlm.nih.gov/geo/). We found several GSH regulatory genes whose expression seems to display a circadian rhythm. We then examined the mRNA expression pattern of several candidates during a 24-h period in the mouse mesencephalon and serum-shocked SH-SY5Y cells using qRT–PCR. The mRNA expression patterns of all of the candidate genes turned out to be constitutive (Supplementary Figs 5 and 6, Supplementary Table 1).

**Figure 2:**
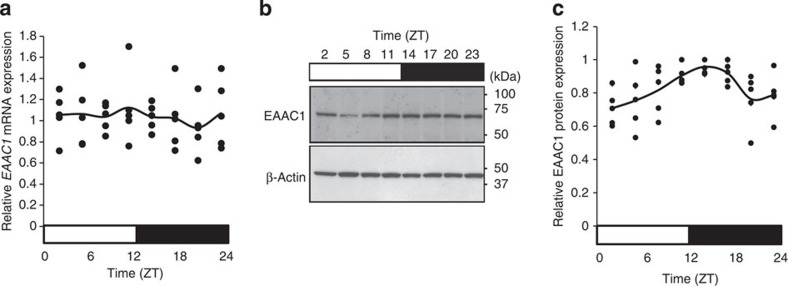
The expression of EAAC1 in the mouse mesencephalon exhibits a diurnal rhythm at the translational but not transcriptional level. (**a**) The profile of *EAAC1* mRNA expression normalized by *Gapdh* expression over a 1-day period in mesencephalons examined by qRT-PCR. The bar below the graph indicates the light (white) and dark (black) periods. The data represent mean values and individual data points obtained from five independent experiments. No significant rhythmicity was detected (*P*=0.54). (**b**) Immunoblots of EAAC1 and β-actin are shown. Molecular weight markers are depicted to the right. Full-length blots are presented in Supplementary Fig. 12. (**c**) Quantification of the data in panel **b** by densitometry. Data represent mean values and individual data points obtained from five independent experiments and were analysed by one-way ANOVA and cosinor analysis. A significant diurnal change was detected (*P*=0.0051).

There are some genes whose protein levels are rhythmic, although their corresponding mRNA is not rhythmic^16^. We next examined the protein expression of EAAC1, which is known as a transporter for a rate-limiting precursor of GSH synthesis^5^. Interestingly, the protein expression of EAAC1 exhibited a diurnal variation with a peak at ZT14 and a trough at ZT2 in the mesencephalon, although its mRNA expression was arrhythmic (Fig. 2a–c). The amplitude of EAAC1 expression was significantly high, with 1.4-fold changes. This suggests that the expression of EAAC1 is regulated by a post-transcriptional regulation mechanism.

### Diurnal oscillation of miRNAs that target the EAAC1 3′-UTR

**Figure 3:**
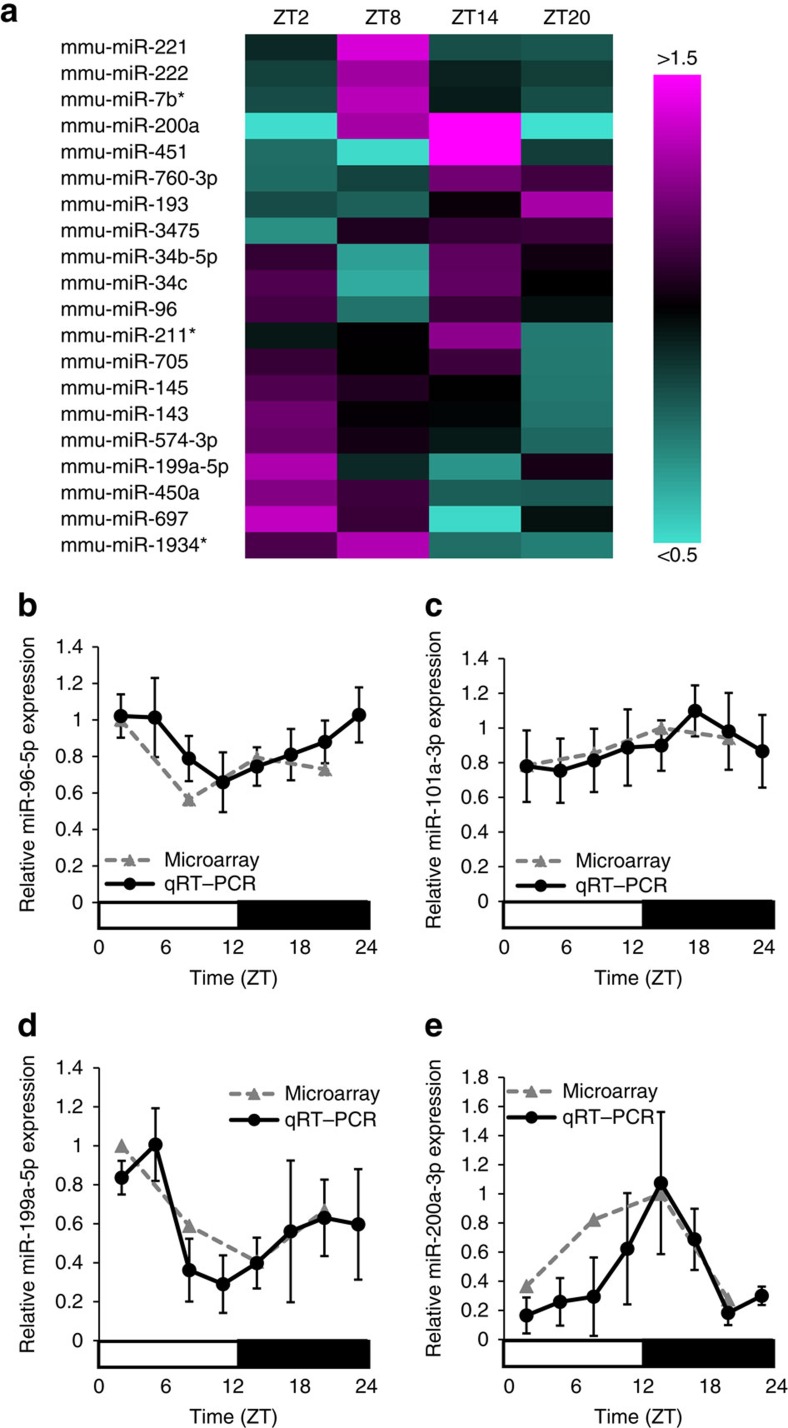
List of EAAC1-targeting miRNAs that exhibit diurnal rhythm. (**a**) We analysed the pattern of miRNA expression over 24 h in the mesencephalon using a miRNA microarray. A heat map of EAAC1-targeting miRNAs with diurnal expression pattern is shown. The heat scale at the right of the panel represents a linear scale, with magenta, black and turquoise representing high, average and low expression, respectively; Expression profiles of miR-96-5p (**b**), miR-101a-3p (**c**), miR-199a-5p (**d**) and miR-200a-3p (**e**) over 24 h in mesencephalon examined by a microarray or qRT-PCR. Data represent mean values ±s.e.m. obtained from eight independent experiments and were analysed by a one-way ANOVA and cosinor analysis. Significant rhythmicity of the expression patterns of miR-96-5p and miR-199a-5p miR-200a-3p was revealed (*P*=0.0082, 0.026 and 0.023, respectively) but not of miR-101a-3p (*P*=0.053).

It was shown that post-transcriptional control could be exerted by miRNAs, which are reported to be crucial modulators of gene expression^14^. To identify the miRNAs that regulate the diurnal rhythm of EAAC1 expression, we first screened miRNAs with a diurnal expression pattern using a miRNA microarray. We identified 20 miRNAs with diurnal oscillations (more than 1.5-fold changes) (Fig. 3a) and 106 miRNAs with lower amplitude (1.2–1.5-fold changes) (Supplementary Fig. 7).

Among the candidates with diurnal oscillations, our computational analysis of candidate miRNA prediction using established programmes revealed three miRNAs-miR-96-5p, miR-199a-5p and miR-200a-3p-as possible candidates, with oscillations that target the 3′-UTR of human, mouse and rat EAAC1. The sequences of these miRNAs’ target sites are highly conserved among these animals. We also identified miR-101a-3p as a conserved EAAC1-targeting miRNA candidate, although this miRNA is classified among the lower-amplitude candidates (Supplementary Fig. 7).

Using qRT–PCR, we confirmed that miR-96-5p, miR-199a-5p and miR-200a-3p oscillate in a diurnal manner as shown by the microarray data (Cosinor; *P*=0.0082, 0.026 and 0.023, respectively) but that miR-101a-3p does not (Cosinor; *P*=0.053). The miR-96-5p levels reached a maximum at ZT23 and minimum at ZT11, with 1.6-fold amplitude (Fig. 3b). MiR-199a-5p and miR-200a-3p had especially large amplitudes, with more than threefold changes and peaks at ZT5 and ZT14, respectively (Fig. 3d,e). Our statistical analysis for rhythmicity revealed that miR-101a-3p had no significant rhythm, although analysis of variance (ANOVA) revealed significant variation (ANOVA; *P*=0.032, Cosinor; *P*=0.053), suggesting that miR-101a-3p is needed for temporal regulation during a day (Fig. 3c). These oscillations of miRNAs such as miR-96-5p, miR-199a-5p and miR-200a-3p are unique in mature miRNA processing; other miRNAs such as miR-101a-3p exhibit no significant diurnal changes in expression (Supplementary Fig. 8).

### Effect of miRNAs on EAAC1 and GSH levels

**Figure 4:**
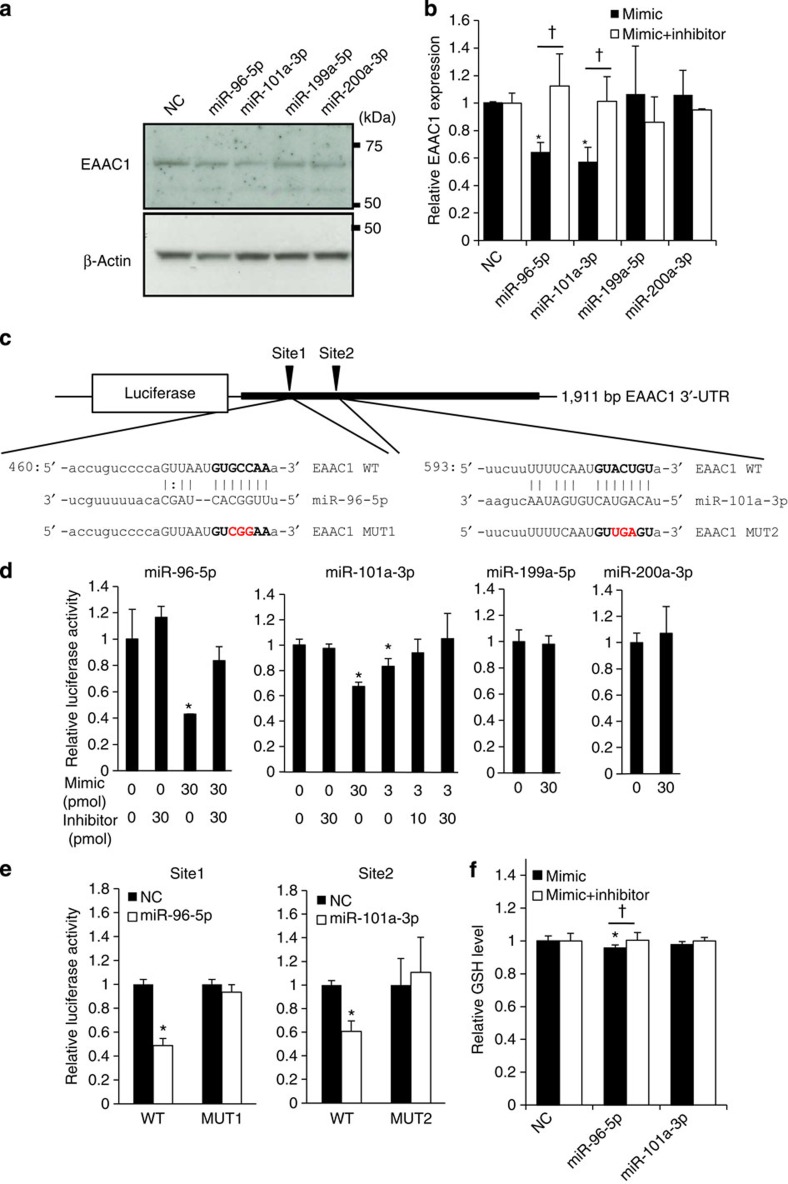
The effect of miRNA transfection on endogenous EAAC1 expression, GSH level and luciferase reporter gene assay using EAAC1 3′-UTR. (**a**) The endogenous expressions of EAAC1 and β-actin in HEK293 cells transfected with each miRNA mimic are shown. NC represents a negative control mimic that has been proven to have no target in human genes. Molecular weight markers are depicted to the right. Full-length blots are presented in Supplementary Fig. 13. (**b**) Quantification of the data in **a** and Supplementary Fig. 14 by densitometry is shown. Data represent mean values±s.e.m. obtained from six independent experiments and were analysed by Steel’s test. *P<0.05 relative to negative control. †*P*<0.05, effect of miRNA inhibitor. (**c**) A schematic plot of the luciferase constructs of human EAAC1 3′-UTR. The sequences for the target site of miR-96-5p and miR-101a-3p on EAAC1 3′-UTR region are shown. Mutation was added in a core sequence (bold font) of each miRNA target site on EAAC1 3′-UTR (red font). (**d**) Relative luciferase activity in SH-SY5Y cells transfected with luciferase constructs in **c** with miRNA mimic (3 pmol or 30 pmol) or inhibitor (10 pmol or 30 pmol) are shown (*n*=6 for each condition). Data represent mean values ±s.e.m. and were analysed by Williams’ test. *P<0.025 relative to negative control. (**e**) Effects of mutation in target sites of miR-96-5p and miR-101a-3p on luciferase activity (*n*=6 for each condition). Data are mean values ±s.e.m. and were analysed by Student’s *t*-test. *P<0.05 relative to negative control. (**f**) Effects of miR-96-5p and miR-101a-3p on the GSH level in HEK293 cells (*n*=10 for each condition). Data are mean values ±s.e.m. and were analysed by Steel’s test. *P<0.05 relative to negative control. †*P*<0.05, the effect of the miRNA inhibitor.

We next examined whether the identified miRNAs regulate GSH levels and EAAC1 expression, using HEK293 cells. We took advantage of the fact that among the EAAT family only EAAC1 is expressed, and that cystine uptake is almost negligible in this cell line^26^. We first investigated the effect of miRNA transfection on EAAC1 expression. The transfection of miR-96-5p or miR-101a-3p into HEK293 cells decreased EAAC1 protein compared with control miRNA (Steel’s test; *P*<0.05), whereas miR-199a-5p and miR-200a-3p had no effects on protein expression (Fig. 4a,b).

We then tested miR-96-5p and miR-101a-3p as EAAC1-targeting miRNAs, using a luciferase reporter assay. We made constructs of human or mouse EAAC1 3′-UTR cloned into a luciferase reporter plasmid (Fig. 4c, Supplementary Fig. 9a). Consistent with the decreased endogenous expression of EAAC1 by these miRNAs, the luciferase activity was significantly lower when miR-96-5p or miR-101a-3p was transfected (Williams’ test; *P*<0.025) (Fig. 4d, Supplementary Fig. 9b). This reduction was blocked by the miRNA inhibitors. Mutation of the core sequence on the EAAC1 3′-UTR target site also blocked the reduction of luciferase activity by miRNAs (Fig. 4e). In contrast, miR-199a-5p and miR-200a-3p had no effect on the luciferase activity, which is also consistent with the western blotting results (Fig. 4a,d, Supplementary Fig. 9b).

Finally, we examined whether miR-96-5p and miR-101a-3p affect the GSH level. We transfected miR-96-5p or miR-101a-3p into HEK293 cells and then measured the relative GSH levels. The result showed that the GSH level was significantly decreased by miR-96-5p (Steel’s test; *P*<0.05) but not by miR-101a-3p, suggesting that miR-96-5p regulates GSH levels through EAAC1 3′-UTR (Fig. 4f).

Here are some dissociations between the miRNAs’ effect on EAAC1 and that on the GSH level. The miR-96-5p caused a much larger change in EAAC1 than in the actual GSH level. miR-101a-3p had no effect on the GSH level, whereas it markedly decreased the EAAC1 level. Our unpublished data revealed that GTRAP3-18 (also known as Addicsin), an inhibitory protein of EAAC1, is reduced by the transfection of miR-101a-3p, suggesting that the bilateral negative effects for EAAC1 and GTRAP3-18 or other inhibitory factors are counteracted so that the change in the GSH level is less than expected.

### Increased GSH and EAAC1 levels by miR-96-5p inhibitor

**Figure 5:**
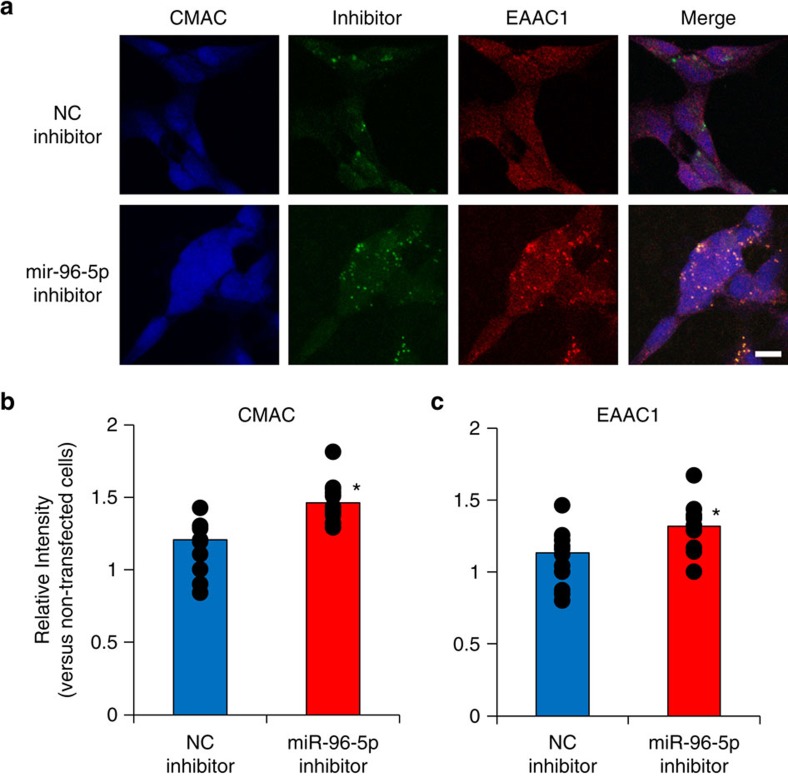
Effect of miR-96-5p inhibitor transfection on GSH level and EAAC1 expression in SH-SY5Y cells. (**a**) Confocal images showing the effect of miR-96-5p inhibitor transfection or negative control (NC) inhibitor (green) on the intensity of CMAC as a marker of GSH (blue) and EAAC1 expression (red). The miR-96-5p inhibitor increased the CMAC and EAAC1 levels compared with the NC inhibitor. Scale bar, 10 μm. (**b**,**c**) The density of CMAC (**b**) and EAAC1 level (**c**) (*n*=11; 10–20 cells were measured in each sample). Data are mean values and individual data points and were analysed by Student’s *t*-test. *P<0.05 relative to NC inhibitor. The number of individual data points is the same as the sample size, although some points overlap.

We next investigated the role of endogenous miR-96-5p in SH-SY5Y cells. We transfected miR-96-5p inhibitor and measured the GSH level using same method as in Fig. 4f. No significant change was detected, however (Supplementary Fig. 10), probably because of the low transfection efficiency (Supplementary Fig. 4a). To evaluate the intracellular GSH level, we used 7-amino-4-chloromethylcoumarine (CMAC), which fluoresces upon conjugation with GSH after the transfection of the fluorescein-labelled miR-96-5p inhibitor or the negative control inhibitor. As shown in Fig. 5a,b, a significantly higher intensity of CMAC was observed in the cells transfected with miR-96-5p inhibitor compared with the negative control or non-transfected cells (Student’s *t*-test; *P*<0.05). The expression of EAAC1 was also increased in the cells transfected with miR-96-5p inhibitor (Student’s *t*-test; *P*<0.05) (Fig. 5c), suggesting that the miR-96-5p inhibitor increased the expression of EAAC1 and GSH level by blocking endogenous miR-96-5p in SH-SY5Y cells. Similar results were obtained using HEK293 cells (Supplementary Fig. 4b,c), namely, the transfection of miR-96-5p inhibitor in HEK293 cells increased the cell viability, GSH level and EAAC1 level 18 or 24 h after the serum shock when the GSH level and protective activity against oxidative stress were lowest. These results suggest that the miR-96-5p inhibitor has a protective role for oxidative stress by increasing EAAC1 and GSH levels in cultured cells.

### Prevention of neurotoxicity by i.c.v. miR-96-5p inhibitor

**Figure 6:**
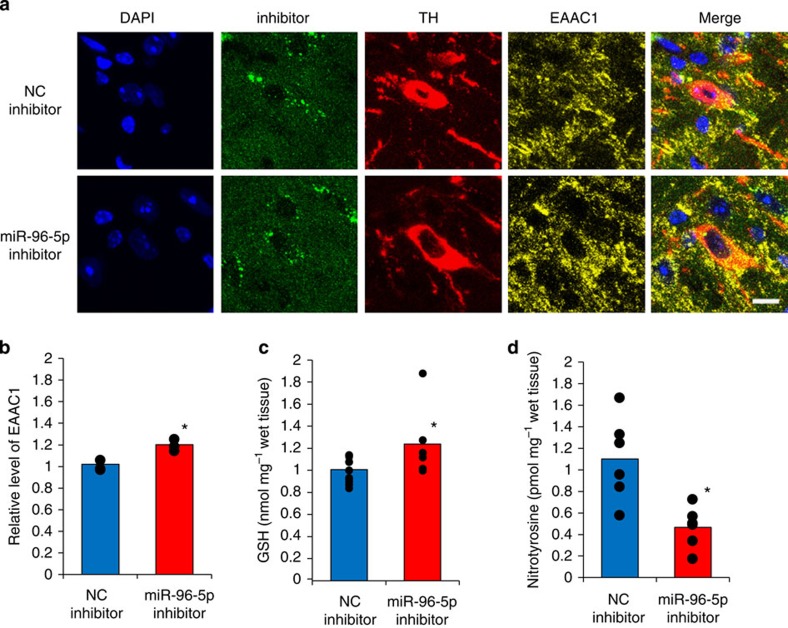
Effect of the i.c.v. administration of miR-96-5p inhibitor on the GSH level, EAAC1 expression and neuroprotection *in vivo.* (**a**) Confocal images showing the effect of miR-96-5p inhibitor or negative control (NC) inhibitor (green) administration on EAAC1 expression (yellow). The inhibitors were administered i.c.v. for 1 week. The brains were then fixed at ZT5 when the lowest GSH amount was observed, and were sagittally sliced. Tyrosine hydroxyrase (red) was used for the dopaminergic neurons marker. The nuclei were stained with DAPI (blue). The miR-96-5p inhibitor increased the amount of EAAC1 compared with the NC inhibitor. Scale bar, 10 μm. (**b**) The density of EAAC1 expression of TH-positive neurons (*n*=3; 50–100 neurons per sample). Data are mean values and individual data points and were analysed by Student’s *t*-test. *P<0.05 relative to NC inhibitor. (**c**) The GSH levels in mesencephalon after the administration of NC or miR-96-5p inhibitor (NC inhibitor; *n*=10, miR-96-5p inhibitor; *n*=6). Data are mean values and individual data points. The data were analysed by Student’s *t*-test. *P<0.05 relative to the NC inhibitor. (**d**) The level of nitrotyrosine expression in mesencephalon with the injection of either miR-96-5p inhibitor or NC inhibitor. Data are mean values and individual data points and were analysed by Student’s *t*-test. *P<0.05 relative to the NC inhibitor. The number of individual data points is the same as the sample size although some points overlap.

To determine whether miR-96-5p regulates the GSH level via EAAC1 *in vivo*, we administered an i.c.v. injection of miR-96-5p inhibitor to block endogenous miR-96-5p miRNA function. We confirmed that the administered miR-96-5p inhibitor reached the TH-positive dopaminergic neurons in the SNc of the mesencephalon (Fig. 6a). Treatment with miR-96-5p inhibitor for 1 week significantly increased the expression of EAAC1 (Student’s *t*-test; *P*<0.05) (Fig. 6b). In addition, the amount of GSH in the mesencephalon at ZT5, when the lowest GSH levels were observed, was also increased compared with the negative control inhibitor injection (Student’s *t*-test; *P*<0.05) (Fig. 6c). This increased amount of GSH was almost equal to the level at ZT17, when highest GSH concentration was observed (Figs 1a and 6c), indicating that the miR-96-5p inhibitor increased the GSH levels by increasing the EAAC1 expression.

There is another possibility; that is, the miR-96-5p inhibitor affects core clock components, and that is why the GSH level seems to be increased with the miR-96-5p inhibitor injection as a result of a phase shift even under the LD cycle. We performed western blotting and detected PER1, PER2, BMAL1 and CLOCK. No significant change was observed at ZT5 or ZT17 (Supplementary Fig. 11).

Next, we determined whether the miR-96-5p inhibitor has a protective role against oxidative stress *in vivo*. After the i.c.v. injection of either the miR-96-5p inhibitor or a negative control inhibitor, we took mesencephalic slices containing SNc and treated them with SIN-1, generating nitric oxide, which reacts with superoxide to produce peroxynitrite, a potent toxic oxidating/nitrating agent. Nitrotyrosine is a permanent marker of peroxynitrite attack on proteins, revealing oxidative/nitrosative stress damage; treatment with SIN-1 thus increases nitrotyrosine expression^27^. As shown in Fig. 6d, the nitrotyrosine levels in the mesencephalon with the injection of the miR-96-5p inhibitor were significantly lower compared with the negative controls.

These results suggest that miR-96-5p inhibitor plays a neuroprotective role in the regulation of GSH levels via EAAC1 expression in the SNc.

## Discussion

In this study we found that miR-96-5p could be a regulator of the GSH level via EAAC1 to control the ROS level in the SNc. ROS play an important role in a variety of physiological systems, including the regulation of autophagy, immunity and differentiation^28^. They also act as signalling molecules for cell proliferation, neurogenesis and circadian rhythm^29^. These metabolic pathways requiring ROS in rodents proceed mostly during the daytime^19^,^30^,^31^,^32^.

Interestingly, it seems that ROS production also tends to be under circadian control with peak levels during the day^33^. As the overproduction or misregulation of ROS leading to oxidative stress states causes several diseases such as neurodegenerative diseases, there must be the regulators for the cycles of ROS build up and elimination. One of these regulators is GSH, which acts as an antioxidant against any form of ROS. Several reports have shown that a circadian clock regulates the GSH level and its enzymes in various organisms^34^,^35^,^36^,^37^. Here, we demonstrated that GSH levels also exhibit a diurnal rhythm in the brain, mesencephalon and SCN, reaching a peak during the night, and hitting a trough in the morning (Fig. 1a and Supplementary Fig. 1). The GSH rhythm is anti-phasic, with the rhythm of metabolic events requiring ROS^19^,^30^,^31^,^32^, suggesting that rhythmic GSH regulates the diurnal rhythm of ROS activity.

Based on the finding that H_2_O_2_ is a form of ROS and physiologically generated^38^, it appears that the effect of H_2_O_2_ would be a reflection of the intracellular antioxidant level. The percentage of viable cells after treatment with H_2_O_2_ is time-dependently correlated with the GSH rhythm, suggesting that GSH is a main determinant of the time-dependent protective activity against oxidative stress in dopaminergic cells and also in other types of cells (Fig. 1d,e, Supplementary Fig. 4b,c). Our results demonstrate that the amplitude of the change in the GSH level is ~10–20%, which is surprising because a 30–40% decrease in the GSH level in the SNc of PD patients has been reported from several postmortem studies^4^,^39^,^40^. Basically, the system of diurnal rhythm could be considered to contribute to the efficient use of energy in the human body so that the diurnal oscillation of GSH might be necessary for accelerating the physiological events that need ROS, as well as for minimizing the damage by oxidative stress.

For decades, researchers have studied the molecular circadian clock system composed of a transcriptional/translational feedback loop. In 2005, however, a surprising non-transcriptional circadian system was discovered in cyanobacteria^41^,^42^. This non-transcriptional system may also drive circadian rhythms in mammals, although its mechanism is still unclear^43^. Here we demonstrated that EAAC1 expression displays diurnal patterns at the translational level despite it being expressed constitutively at the transcriptional level, which is seemingly driven by a non-transcriptional circadian system.

We also found that the diurnal expression of EAAC1 is regulated by post-transcriptional regulators, miRNA (Figs 4, 5, 6). The latest miRbase (Release 20) reported 30,424 mature miRNA products; 24,521 precursor hairpin miRNAs have been found so far, and the number of miRNAs is still increasing. Since the expression profile of miR-96-5p exhibits a diurnal rhythm (Fig. 3b), we searched 5 kb upstream of the miR-96-5p encoding region, but found no important cis-element (E-box and RORE sequences) for circadian regulation. Thus, miRNA-processing genes such as Dicer, Drosha and Ago2 may play an important role in composing the circadian rhythm of miRNA. We infer miRNA to be one of the most important components in the non-transcriptional circadian system.

Finally, we administered i.c.v. injections of a miR-96-5p inhibitor and observed an increase in the GSH level along with an increased EAAC1 expression in the TH-positive neurons of the mouse SNc (Fig. 6). Moreover, the injection of the miR-96-5p inhibitor involves a protective effect against oxidative stress by SIN-1.

These results are quite interesting in that the miR-96-5p inhibitor injection could be a therapeutic agent for increasing GSH levels in the brain. It has not been thought possible to increase GSH levels in the brain through the extracellular administration of GSH itself or its precursor cysteine^5^. Thus, at present, *N*-acetyl-L-cysteine is the only effective agent for increasing GSH levels with low toxicity in order to increase the lifespan of an ageing mouse model^9^,^44^. MiR-96-5p inhibitors could be another option. Further research is needed to confirm this possibility.

## Methods

### Animals

Adult male ddY mice (8–10 weeks old) were maintained in a light/dark (LD) 12 h/12 h cycle. The time of ‘lights on’ is defined as the zeitgeber time (ZT) 0 and the time of ‘lights off’ is defined as ZT12. All mice were starved for 1 day and then perfused intracardially with phosphate-buffered saline (PBS) under CO_2_ anaesthesia. Tissues were collected at ZT 2, 5, 8, 11, 14, 17, 20 and 23. For the dissection of the SCN area, a pyramid of the anterior hypothalamus was dissected out from the ventral surface of the hypothalamus. For the dissection of the SNc area, the brain was cut in half sagittally, and then the area of the mesencephalon was dissected out. All animal protocols were approved by the Animal Experimentation Committee of the Teikyo University School of Medicine.

### Serum shock

SH-SY5Y and HEK293 cells were grown in Dulbecco’s modified Eagle’s medium or minimum essential medium (Life Technologies, Frederick, MD, USA), respectively, supplemented with 10% fetal bovine serum and 1% penicillin–streptomycin at 37 °C under 5% CO_2_. Cells were starved 1 day before serum shock and then changed to a medium containing 50% horse serum and incubated for 2 h, after which the serum-rich medium was replaced with medium containing 10% serum. The time just after the serum shock is defined as time =0.

### Detection of GSH levels

Brain tissue was homogenized in a 10-fold volume of GSH extraction buffer (5% trichloroacetic acid and 5 mM EDTA) and centrifuged at 1,200*g* for 15 min at 4 °C. Supernatants were used for measurements. Tissue GSH was detected with 4-fluoro-7-sulfamoylbenzofurazan (Dojindo, Kumamoto, Japan), a fluorogenic labelling reagent for thiols, as described previously^45^,^46^. The LC-20AD liquid chromatography system (Shimadzu, Kyoto, Japan) was used for GSH detection. An analytical column, Inertsil ODS-2 (150 × 4.6 mm ID 5 μm) (GL Sciences, Torrance, CA, USA), was fixed at 40 °C and connected through a corresponding guard column (10 × 4.0 mm ID 5 μm; GL Sciences). A stepwise gradient elution was programmed with solvents A (50 mM potassium biphthalate at pH 4.0) and B (8% acetonitrile in solvent A). The mobile phase was held at 80% solvent A and 20% B for 6 min, followed by a 10-min programme held at 100% solvent B. The flow rate of the eluate was 1.0 ml min^−1^. All samples were injected into the column with an Auto Injector (Shimadzu). An RF-530 fluorescence spectrometer (Shimadzu) was used with excitation and emission at 380 nm and 510 nm, respectively. The signals from the detector were recorded on a Chromatopac C-R4A (Shimadzu). Tissue GSH concentrations were calculated from the peak area standardized with known amounts of GSH.

For the measurement of GSSG concentration, DTNB-GSSG reductase recycling assay was performed using glutathione quantification kit (Dojindo) according to the manufacturer’s protocol. The ratio of GSSG to GSH was calculated as dividing GSSG concentration by GSH concentration.

We determined the GSH concentrations in the SH-SY5Y and HEK293 cells by using ThioGlo-1 (Merck, Darmstadt, Germany), a maleimide reagent that produces a highly fluorescent adduct upon reaction with thiol groups. The GSH content was estimated from the fluorescence response via the interaction of ThioGlo-1 mainly with intracellular GSH. Cells were incubated at 37 °C for 30 min with 10 μM ThioGlo-1, and the level of fluorescence was measured using a Multimode Detector DTX800 (Beckman Coulter, Indianapolis, IN, USA). To remove the effect of the cystine transport system, we added 100 μM DTT at 1 day before harvesting.

### Viable cell count

After the treatment of SH-SY5Y cells with 500 μM H_2_O_2_ or HEK293 cells with 5 mM H_2_O_2_ for 2 h, we suspended the cells with 0.5 ml PBS and placed them in a 1.5-ml tube. Next, we added 0.1 ml of 0.4% Trypan Blue and incubated the cells for 5 min. A haemocytometer was filled with cell suspensions, and at least 100 cells were counted under a microscope. Blue colour-stained and non-stained cells were considered damaged and viable cells, respectively.

### Quantitative RT-PCR

RNA isolation was carried out using Trizol Reagent (Life Technologies). For mRNA quantification, we conducted reverse transcription (RT) on all individual RNA samples using High-Capacity cDNA Reverse Transcription Kits (Life Technologies) with random hexamers as the RT primers, according to the manufacturer’s protocol. Real-time PCRs were performed using the Light Cycler 330 (Roche, Mannheim, Germany), and the amplifications were done using the SYBR Premix Ex Taq II (Takara, Shiga, Japan). Primers for quantitative RT-PCR were designed using the Primer3Plus software and purchased from Nihon Gene Research Laboratories Inc. (Miyagi, Japan). The list of the primers is presented in Supplementary Table 2.

For the miRNA quantification, we used the miRCURY LNA Universal RT microRNA PCR kit (Exiqon, Vedbaek, Denmark) for RT–PCR reactions. Real-time PCR for miRNA was performed using PCR primer sets (Exiqon) and SYBR Green master mix (Exiqon) on the LightCycler 480 II (Roche) according to the manufacturer’s protocol.

### MiRNA microarray

A pool of five RNA samples extracted from the mouse mesencephalon at each time point (ZT2, 8, 14 and 20) was analysed with the SurePrint G3 Mouse miRNA microarray, protocol version 2.2 (Agilent, Santa Clara, CA, USA) by Hokkaido System Science Co. (Sapporo, Japan). The platform is based on Sanger miRBase version 16.0, and the number of miRNAs on the chip is 1,055.

Scanned microarray raw data were imported into GeneSpring Gx (Agilent) and then normalized to the 75th percentile per chip. Data in which the signal value was three times more than the error value were chosen for the data analysis. The fold changes of ZT2 versus ZT8, ZT2 versus ZT14, ZT2 versus ZT20, ZT8 versus ZT14, ZT8 versus ZT20, and ZT14 versus ZT20 were used for listing the miRNA candidates.

For the prediction of candidate miRNA that target the 3′-UTR of human, mouse and rat EAAC1, we used eight established programs that are Diana-microT, miRanda, miRDB, miRWalk, RNAhybrid, PICTAR, PITA and TargetScan.

### Western blotting

The amount of protein was determined using the BCA protein assay (Thermo Scientific, Rockford, IL, USA), and the same amounts of proteins were normalized for total protein. The protein samples were boiled in RIPA buffer (20 mM Tris–HCl (pH 7.5), 150 mM NaCl, 1% NP-40, 1% sodium deoxycholate, 0.1% sodium dodecyl sulphate (SDS) and protease inhibitor cocktail; Sigma-Aldrich, St Louis, MO, USA), separated by SDS–polyacrylamide gel electrophoresis (PAGE), and transferred to polyvinylidene fluoride (PVDF) membranes (Bio-Rad, Hercules, CA, USA). Non-specific binding was blocked with 5% skim milk in PBS-Tween20, and proteins were probed with anti-EAAC1 (Abcam, Cambridge, MA) at 1:1,000 dilution, anti-PER1 (Abcam) at 1:200, anti-PER2 (Abcam) at 1:1,000, anti-BMAL1 (Abcam) at 1:1,000, anti-CLOCK (Abcam) at 1:1,000 and anti-β-actin (Sigma-Aldrich) at 1:10,000 dilution. After a wash with PBS-Tween20, the horseradish peroxidase-labelled secondary antibodies were probed and detected with the ECL prime HRP detection kit (GE Healthcare, Piscataway, NJ, USA). We performed the quantification of the EAAC1 level using a serial dilution of ZT14 samples as the standard curve.

### Luciferase reporter assays of miRNA targeting

The 3′-UTR of human EAAC1 (NM_004170) or mouse EAAC1 (NM_009199) containing five potential target sites for miR-96-5p, miR-101a-3p, miR-199a-5p and miR-200a-3p were amplified from cDNA of SH-SY5Y cells using forward primer 5′-GGGAGCTCATAGGCCGGCCCCTGGCTGCAGATG-3′ and reverse primer 5′-GCACGCGTCTATGCCGAAAGAATGAGGGAAGTGTT-3′ or mouse mesencephalon using forward primer 5′-GGGAGCTCATAGGCCATGCCTGACCTCAGATTGA-3′ and reverse primer 5′-GCACGCGTCTATGCCTAAGGGGAGAAAGAGTGGG-3′, respectively. PCR products amplified with Prime STAR HS (Takara) were cloned into the pMD20 T-vector using Mighty TA-cloning kit (Takara) and then confirmed by DNA sequencing (FASMAC, Atsugi, Japan). These inserts were then removed from pMD20-T vector by SacI/MluI digestion (human EAAC1 3′-UTR) or SpeI/MluI digestion (mouse EAAC1 3′-UTR) and then subcloned into the firefly luciferase reporter vector, pMIR-REPORT (Promega, Madison, WI, USA). The mutation of the miRNA target sequence on EAAC1 3′-UTR was performed using the Mutagenesis kit (Takara). The primer sequences for mutagenesis were as follows: site1; 5′-TAATGTCGGAAAATGTCAATTTTTAAC-3′ (forward) and 5′-CATTTTCCGACATTAACTGGGGACAGG-3′ (reverse), site2; 5′-CAATGTTGAGTATTGGGACGCTGGTAA-3′ (forward) and 5′-CAATACTCAACATTGAAAAAAGAATCC-3′ (reverse). Cells were transfected with the appropriate combination of pMIR-human EAAC1 3′-UTR or pMIR-mouse EAAC1 3′-UTR constructs with *Renilla* luciferase vector (pRL), miRNA mimic and miRNA inhibitor using Lipofectamine RNAiMax (Life Technologies). Firefly luciferase activity was normalized to *Renilla* luciferase activity. Luciferase activity was measured by a Dual-luciferase Reporter Assay System (Promega) using a luminometer (Turner Biosystems/Promega).

### Immunocytochemistry

We used the chloromethyl reagent 7-amino-4-chloromethylcoumarine (CMAC) (Life Technologies), which produces a highly fluorescent adduct upon reaction with thiol groups for the evaluation of intracellular GSH in SH-SY5Y cells. Cells were incubated at 37 °C for 15 min with 5 μM CMAC and then incubated with serum-free media for 30 min. The cells were fixed with 4% PFA and then permeabilized with 0.05% Triton-X100 in the case of multiple staining with EAAC1. Non-specific staining was blocked with the reagent PBS containing 1% BSA/0.2% TritonX-100, and the cells were then incubated with anti-EAAC1 (Abcam) at 1:1,000 dilution overnight at 4 °C. After a wash with PBS-Tween20, the cells were labelled with fluorescent-labelled secondary antibodies. Finally, the cells were mounted using a Fluoromount-Plus (Diagnostic Biosystems, Pleasanton, CA, USA) and captured with a Nikon A1 confocal microscope.

### Intracerebroventricular injections

The miR-96-5p inhibitor or negative control inhibitor (Exiqon) dissolved in artificial cerebrospinal fluid (aCSF) containing 130 mM NaCl, 3.5 mM KCl, 1.25 mM NaH_2_PO_4_, 2 mM MgSO_4_, 2 mM CaCl_2_, 20 mM NaHCO_3_ and 10 mM glucose (pH 7.4) was administered into the right lateral ventricle of mice. The intraventricular injections were made under stereotaxic guidance. As described previously^47^, a hole was made 0.3 mm caudal to the bregma, 1.2 mm from the midline and the syringe needle tip was lowered 2.5 mm below the dura. Next, 100 μl of 3.0 nmol of miR-96-5p inhibitor or negative control inhibitor in aCSF was injected i.c.v. for 1 week.

For the experiment on oxidative stress, the brain was immediately cut into 300-μm-thick slices in gassed (95% oxygen/5% CO_2_) ice-cold aCSF after decapitation. The experiments were initiated by transferring mesencephalic slices to tubes each containing aCSF at 30 °C that was continuously bubbled with 95% oxygen/5% CO_2_. Mesencephalic slices were exposed to 1 mM 3-morpholinosydnonimine (SIN-1, Santa Cruz Biotechnology, Santa Cruz, CA, USA), an NO donor, for 30 min as described previously^27^. The expression of nitrotyrosine was quantified with an OxiSelect nitrotyrosine ELISA kit (Cell Biolabs, San Diego, CA, USA) according to the manufacturer’s instructions.

### Immunohistochemistry

Mice were perfused with PBS containing 4% performaldehyde. Their brains were then placed in an optimal cutting temperature compound and frozen with liquid nitrogen. Sagittal brain sections were cut on a cryostat at 10 μm thickness and stored at −80 °C. The slices were placed in blocking reagent (PBS containing 1% BSA/0.2% TritonX-100/1 mM EDTA) and then incubated overnight at 4 °C with anti-EAAC1 (Alpha Diagnostics, San Antonio, TX, USA) at 1:200 dilution and anti-tyrosine hydroxylase (Millipore) at 1:1,000 dilution. After a wash with PBS-Tween20, the slices were labelled with fluorescent-labelled secondary antibodies, Alexa-Fluor 594 anti-mouse IgG and Alexa-Fluor 647 anti-rabbit IgG at 1:1,000 dilutions. For nuclear labelling, 4′,6-diamidino-2-phenylindole (DAPI) (Dojindo) was used. The section was mounted using a Fluoromount-Plus (Diagnostic Biosystems) and captured with a Nikon A1 confocal microscope.

### Statistics

Data were analysed by one-way ANOVA. The diurnal rhythm assessment was further confirmed using the free Cosinor Periodogram analysis programme ver.2.3 from Circadian Rhythm Laboratory provided by Dr R. Reffinetti to determine the circadian periodicity. For the analysis of miRNA mimic or miRNA inhibitor effects, the appropriate statistical test noted in the figure legends was used. Differences with *P*<0.05 were considered significant.

## Author contributions

C.K. and T.N. designed the experiments and wrote the manuscript. K.A. performed i.c.v. injection, slice cultures and the related data analyses. N.M. performed the experiments using HPLC and the related data analyses. C.K. performed all other experiments and statistical analysis. K.K.U. and M.W. provided experimental supports for *in vivo* and *in vitro* experiments, respectively.

## Additional information

**How to cite this article:** Kinoshita, C. *et al.* Rhythmic oscillations of the microRNA miR-96-5p play a neuroprotective role by indirectly regulating glutathione levels. *Nat. Commun.* 5:3823 doi: 10.1038/ncomms4823 (2014).

## Supplementary Material

Supplementary Information Supplementary Figures 1-14 and Supplementary Tables 1-2

## References

[b1] Henchcliffe C. & Beal M. F. Mitochondrial biology and oxidative stress in Parkinson disease pathogenesis. Nat. Clin. Pract. Neurol. 4, 600–609 (2008). 18978800 10.1038/ncpneuro0924

[b2] Finkel T. & Holbrook N. J. Oxidants, oxidative stress and the biology of ageing. Nature 408, 239–247 (2000). 11089981 10.1038/35041687

[b3] Dringen R. Metabolism and functions of glutathione in brain. Prog. Neurobiol. 62, 649–671 (2000). 10880854 10.1016/s0301-0082(99)00060-x

[b4] Sian J. *et al.* Alterations in glutathione levels in Parkinson’s disease and other neurodegenerative disorders affecting basal ganglia. Ann. Neurol. 36, 348–355 (1994). 8080242 10.1002/ana.410360305

[b5] Aoyama K., Watabe M. & Nakaki T. Regulation of neuronal glutathione synthesis. J. Pharmacol. Sci. 108, 227–238 (2008). 19008644 10.1254/jphs.08r01cr

[b6] Maragakis N. J. & Rothstein J. D. Glutamate transporters: animal models to neurologic disease. Neurobiol. Dis. 15, 461–473 (2004). 15056453 10.1016/j.nbd.2003.12.007

[b7] Rothstein J. D. *et al.* Knockout of glutamate transporters reveals a major role for astroglial transport in excitotoxicity and clearance of glutamate. Neuron 16, 675–686 (1996). 8785064 10.1016/s0896-6273(00)80086-0

[b8] Aoyama K. *et al.* Neuronal glutathione deficiency and age-dependent neurodegeneration in the EAAC1 deficient mouse. Nat. Neurosci. 9, 119–126 (2006). 16311588 10.1038/nn1609

[b9] Berman A. E. *et al.* N-acetylcysteine prevents loss of dopaminergic neurons in the EAAC1-/- mouse. Ann. Neurol. 69, 509–520 (2011). 21446024 10.1002/ana.22162 PMC4096233

[b10] Bass J. Circadian topology of metabolism. Nature 491, 348–356 (2012). 23151577 10.1038/nature11704

[b11] Kondratov R. V. A role of the circadian system and circadian proteins in aging. Ageing Res. Rev. 6, 12–27 (2007). 17369106 10.1016/j.arr.2007.02.003

[b12] Kondratov R. V., Kondratova A. A., Gorbacheva V. Y., Vykhovanets O. V. & Antoch M. P. Early aging and age-related pathologies in mice deficient in BMAL1, the core componentof the circadian clock. Genes Dev. 20, 1868–1873 (2006). 16847346 10.1101/gad.1432206 PMC1522083

[b13] Willison L. D., Kudo T., Loh D. H., Kuljis D. & Colwell C. S. Circadian dysfunction may be a key component of the non-motor symptoms of Parkinson’s disease: insights from a transgenic mouse model. Exp. Neurol. 243, 57–66 (2013). 23353924 10.1016/j.expneurol.2013.01.014 PMC3994881

[b14] Bartel D. P. MicroRNAs: target recognition and regulatory functions. Cell 136, 215–233 (2009). 19167326 10.1016/j.cell.2009.01.002 PMC3794896

[b15] Cheng H. Y. & Obrietan K. Revealing a role of microRNAs in the regulation of the biological clock. Cell Cycle 6, 3034–3035 (2007). 18075311 10.4161/cc.6.24.5106

[b16] Reddy A. B. *et al.* Circadian orchestration of the hepatic proteome. Curr. Biol. 16, 1107–1115 (2006). 16753565 10.1016/j.cub.2006.04.026

[b17] Harraz M. M., Dawson T. M. & Dawson V. L. MicroRNAs in Parkinson’s disease. J. Chem. Neuroanat. 42, 127–130 (2011). 21295133 10.1016/j.jchemneu.2011.01.005 PMC3163813

[b18] Calcutt G. Diurnal variations in rat blood glutathione levels. Naturwissenschaften 54, 120 (1967). 5585835 10.1007/BF00640587

[b19] Filipski E. *et al.* Persistent twenty-four hour changes in liver and bone marrow despite suprachiasmatic nuclei ablation in mice. Am. J. Physiol. Regul. Integr. Comp. Physiol. 287, R844–R851 (2004). 15217787 10.1152/ajpregu.00085.2004

[b20] Blanco R. A. *et al.* Diurnal variation in glutathione and cysteine redox states in human plasma. Am. J. Clin. Nutr. 86, 1016–1023 (2007). 17921379 10.1093/ajcn/86.4.1016

[b21] Lach H., Surowiak J., Dziubek K., Krawczyk S. & Szaroma W. Cosinor analysis of diurnal changes of the reduced glutathione level in the blood, brain, liver and kidneys of mice, induced by ACTH administration. Acta Biol. Hung. 37, 93–100 (1986). 2823515

[b22] Farooqui M. Y. & Ahmed A. E. Circadian periodicity of tissue glutathione and its relationship with lipid peroxidation in rats. Life Sci. 34, 2413–2418 (1984). 6727575 10.1016/0024-3205(84)90430-2

[b23] Baydas G. *et al.* Daily rhythm of glutathione peroxidase activity, lipid peroxidation and glutathione levels in tissues of pinealectomized rats. Neurosci. Lett. 323, 195–198 (2002). 11959418 10.1016/s0304-3940(02)00144-1

[b24] Balsalobre A., Damiola F. & Schibler U. A serum shock induces circadian gene expression in mammalian tissue culture cells. Cell 93, 929–937 (1998). 9635423 10.1016/s0092-8674(00)81199-x

[b25] Akashi M. & Nishida E. Involvement of the MAP kinase cascade in resetting of the mammalian circadian clock. Genes Dev. 14, 645–649 (2000). 10733524 PMC316464

[b26] Watabe M., Aoyama K. & Nakaki T. Regulation of glutathione synthesis via interaction between glutamate transport-associated protein 3-18 (GTRAP3-18) and excitatory amino acid carrier-1 (EAAC1) at plasma membrane. Mol. Pharmacol. 72, 1103–1110 (2007). 17646425 10.1124/mol.107.039461

[b27] Aoyama K. *et al.* Increased neuronal glutathione and neuroprotection in GTRAP3-18-deficient mice. Neurobiol. Dis. 45, 973–982 (2012). 22210510 10.1016/j.nbd.2011.12.016

[b28] Sena L. A. & Chandel N. S. Physiological roles of mitochondrial reactive oxygen species. Mol. Cell 48, 158–167 (2012). 23102266 10.1016/j.molcel.2012.09.025 PMC3484374

[b29] Dickinson B. C. & Chang C. J. Chemistry and biology of reactive oxygen species in signaling or stress responses. Nat. Chem. Biol. 7, 504–511 (2011). 21769097 10.1038/nchembio.607 PMC3390228

[b30] Arjona A. & Sarkar D. K. Circadian oscillations of clock genes, cytolytic factors, and cytokines in rat NK cells. J. Immunol. 174, 7618–7624 (2005). 15944262 10.4049/jimmunol.174.12.7618

[b31] Kochman L. J., Weber E. T., Fornal C. A. & Jacobs B. L. Circadian variation in mouse hippocampal cell proliferation. Neurosci. Lett. 406, 256–259 (2006). 16930842 10.1016/j.neulet.2006.07.058

[b32] Ma D., Panda S. & Lin J. D. Temporal orchestration of circadian autophagy rhythm by C/EBPbeta. EMBO J. 30, 4642–4651 (2011). 21897364 10.1038/emboj.2011.322 PMC3243590

[b33] Kondratova A. A., Dubrovsky Y. V., Antoch M. P. & Kondratov R. V. Circadian clock proteins control adaptation to novel environment and memory formation. Aging (Albany NY) 2, 285–297 (2010). 20519775 10.18632/aging.100142 PMC2898019

[b34] Beaver L. M. *et al.* Circadian regulation of glutathione levels and biosynthesis in *Drosophila melanogaster*. PLoS ONE 7, e50454 (2012). 23226288 10.1371/journal.pone.0050454 PMC3511579

[b35] Xu Y. Q. *et al.* Diurnal variation of hepatic antioxidant gene expression in mice. PLoS ONE 7, e44237 (2012). 22952936 10.1371/journal.pone.0044237 PMC3430632

[b36] Krishnan N., Davis A. J. & Giebultowicz J. M. Circadian regulation of response to oxidative stress in Drosophila melanogaster. Biochem. Biophys. Res. Commun. 374, 299–303 (2008). 18627767 10.1016/j.bbrc.2008.07.011 PMC2553425

[b37] Pablos M. I. *et al.* Rhythms of glutathione peroxidase and glutathione reductase in brain of chick and their inhibition by light. Neurochem. Int. 32, 69–75 (1998). 9460704 10.1016/s0197-0186(97)00043-0

[b38] Jomova K., Vondrakova D., Lawson M. & Valko M. Metals, oxidative stress and neurodegenerative disorders. Mol. Cell Biochem. 345, 91–104 (2010). 20730621 10.1007/s11010-010-0563-x

[b39] Sofic E., Lange K. W., Jellinger K. & Riederer P. Reduced and oxidized glutathione in the substantia nigra of patients with Parkinson’s disease. Neurosci. Lett. 142, 128–130 (1992). 1454205 10.1016/0304-3940(92)90355-b

[b40] Perry T. L., Godin D. V. & Hansen S. Parkinson’s disease: a disorder due to nigral glutathione deficiency? Neurosci. Lett. 33, 305–310 (1982). 7162692 10.1016/0304-3940(82)90390-1

[b41] Tomita J., Nakajima M., Kondo T. & Iwasaki H. No transcription-translation feedback in circadian rhythm of KaiC phosphorylation. Science 307, 251–254 (2005). 15550625 10.1126/science.1102540

[b42] Nakajima M. *et al.* Reconstitution of circadian oscillation of cyanobacterial KaiC phosphorylation *in vitro*. Science 308, 414–415 (2005). 15831759 10.1126/science.1108451

[b43] O’Neill J. S. & Reddy A. B. Circadian clocks in human red blood cells. Nature 469, 498–503 (2011). 21270888 10.1038/nature09702 PMC3040566

[b44] Kondratov R. V., Vykhovanets O., Kondratova A. A. & Antoch M. P. Antioxidant N-acetyl-L-cysteine ameliorates symptoms of premature aging associated with the deficiency of the circadian protein BMAL1. Aging (Albany NY) 1, 979–987 (2009). 20157581 10.18632/aging.100113 PMC2815755

[b45] Aoyama K., Matsumura N., Watabe M. & Nakaki T. Oxidative stress on EAAC1 is involved in MPTP-induced glutathione depletion and motor dysfunction. Eur. J. Neurosci. 27, 20–30 (2008). 18093171 10.1111/j.1460-9568.2007.05979.x

[b46] Matsumura N. *et al.* Anticonvulsant action of indazole. Epilepsy Res. 104, 203–216 (2013). 23219048 10.1016/j.eplepsyres.2012.11.001

[b47] Watabe M., Aoyama K. & Nakaki T. A dominant role of GTRAP3-18 in neuronal glutathione synthesis. J Neurosci. 28, 9404–9413 (2008). 18799673 10.1523/JNEUROSCI.3351-08.2008 PMC6671108

