# Supplementary material for: Rhythmic oscillations of the microRNA miR-96-5p play a neuroprotective role by indirectly regulating glutathione levels
Source: Nat Commun. 2014 May 7;5:3823. doi: 10.1038/ncomms4823 (PMC4024755; doi:10.1038/ncomms4823)
Supplement: Supplementary Information — Supplementary Figures 1-14 and Supplementary Tables 1-2 [file ncomms4823-s1.pdf]

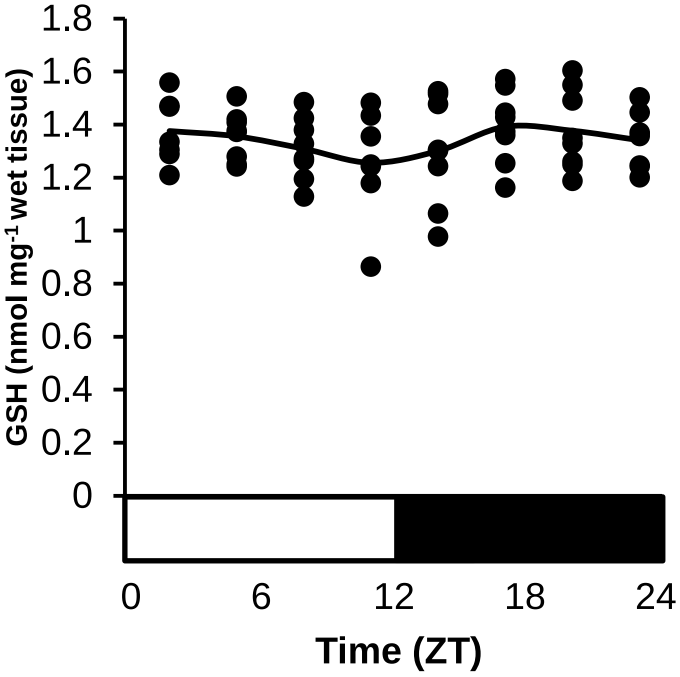

Supplementary Figure 1

Diurnal variation of GSH level in the SCN where core clock is located.

Diurnal changes in GSH levels in the SCN are shown (n=8, each point). The bar below the graph indicates the light (white) and dark (black) periods. Data represent mean values and individual points. Data were analyzed by a one-way ANOVA and cosinor analysis. Statistically significant diurnal rhythm were detected (P=0.037). The number of individual data points is the same as the sample size although some points overlap.

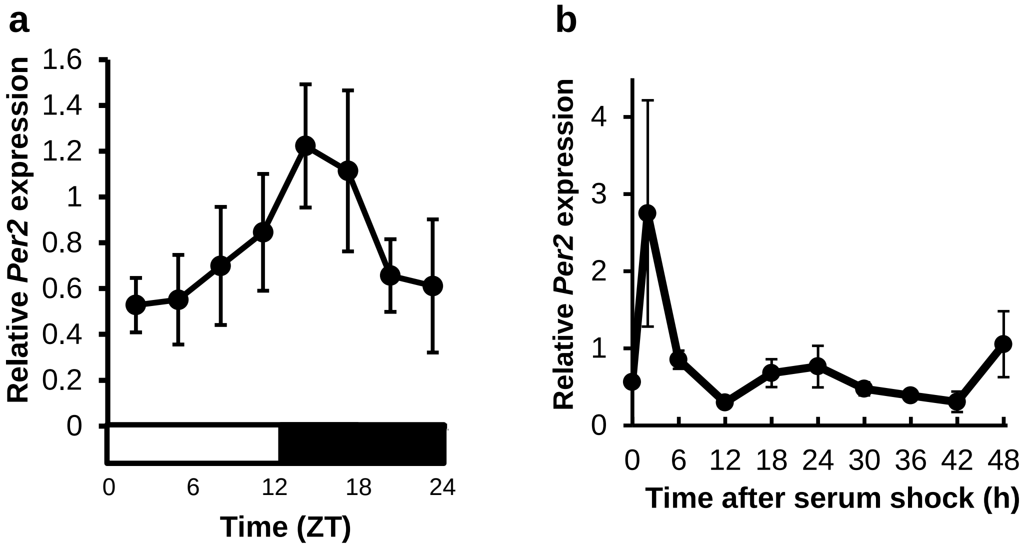

Supplementary Figure 2

Diurnal oscillation of clock gene *Per2* in mesencephalon and serum-shocked SH-SY5Y cells.

(a) The profile of *Per2* expression normalized by *Gapdh* expression over 24 h in mouse mesencephalon examined by qRT-PCR is shown. The bar below the graph indicates the light (white) and dark (black) periods. The data represent mean values ± s.e.m obtained from eight independent experiments and were analyzed by one-way ANOVA and cosinor analysis. A statistically significant diurnal variation was detected (Cosinor; P=0.0006) (b) The profile of *Per2* expression normalized by *Gapdh* expression in SH-SY5Y cells after serum shock is shown (n=4, each points). Data represent mean values ± s.e.m and were analyzed by a one-way ANOVA and cosinor analysis. A statistically significant diurnal variation was detected (Cosinor; P=0.0028).

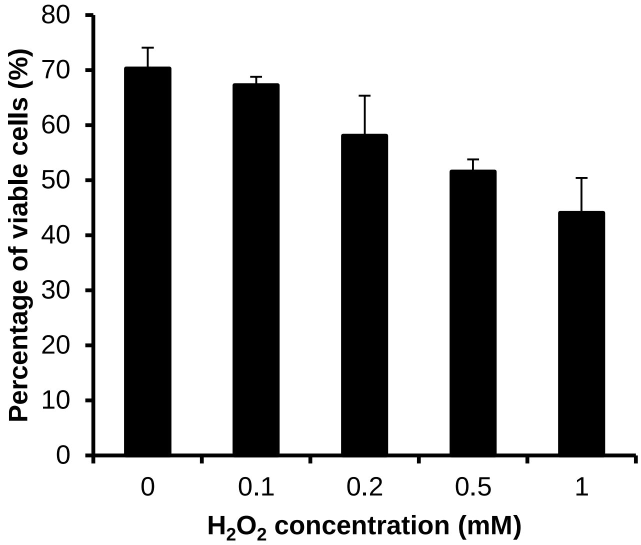

Supplementary Figure 3

Dose-dependent decline in cell viability induced by hydrogen peroxide

Percentages of viable cells after various concentration of H_2O_2 treatment for 2 h are shown. Data represent mean values ± s.e.m obtained from three independent experiments. At least 100 cells were counted for each experiments.

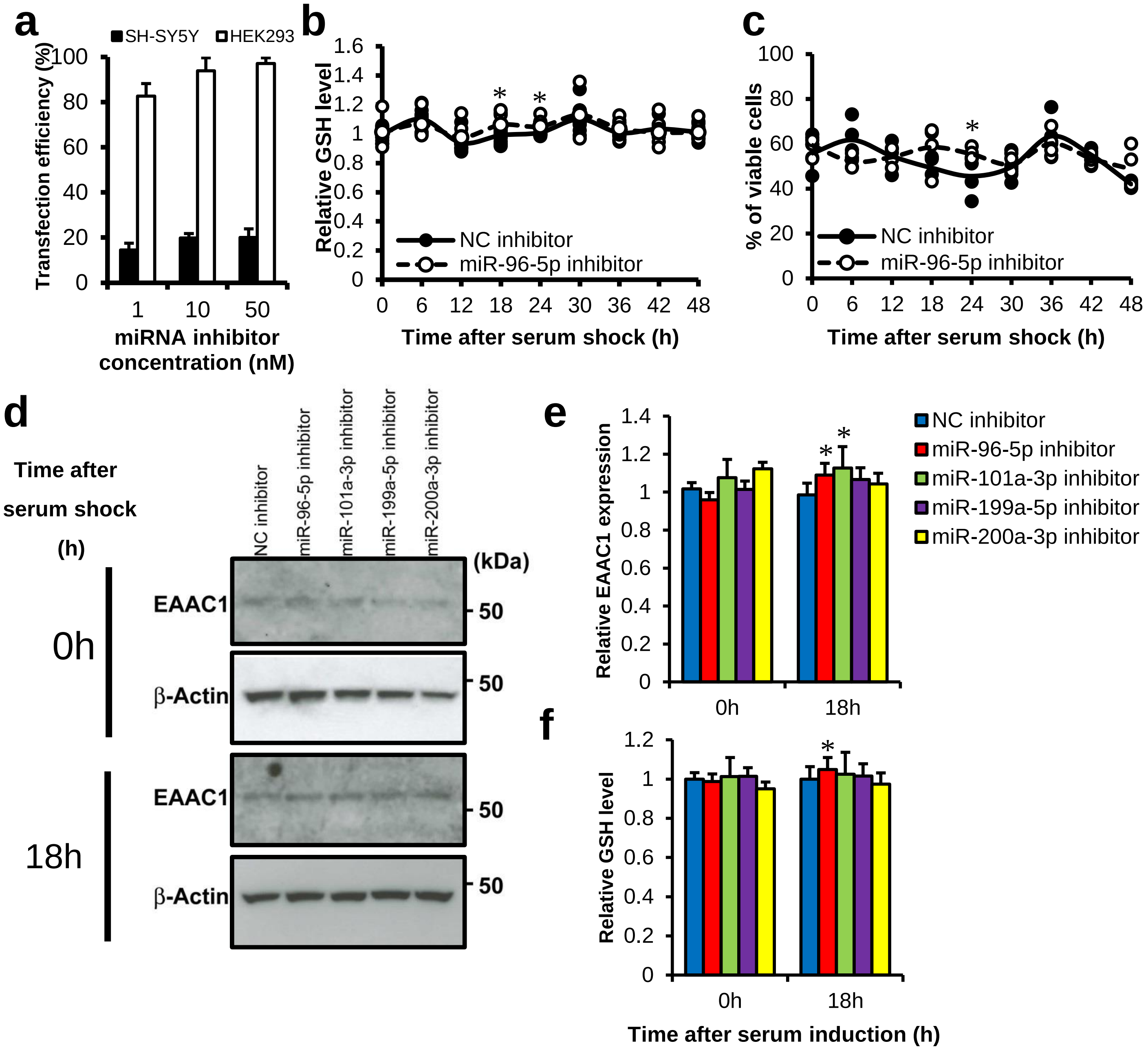

Supplementary Figure 4

Effects of miRNA inhibitor transfection on cell viability, GSH and EAAC1 level in HEK293 cells

(a) Transfection efficiencies of SH-SY5Y or HEK293 cells are shown. Data represent mean values ± s.e.m. (b) Rhythmic changes of GSH levels in serum-shocked HEK293 cells transfected with miR-96-5p inhibitor or negative control (NC) inhibitor are shown. Data represent mean values and individual points. Cosinor analysis represents significant variation in the cells transfected with NC inhibitor (P=0.0011) but not miR-96-5p inhibitor (P=0.055) Significant inhibitor effects were analyzed by Student's t-test. (*P<0.05). The number of individual data points is the same as the sample size although some points overlap. (c) Time-dependent changes of viable cell percentages after treatment of H_2O_2 for 2 h at each time point are shown. Data represent mean and individual data points. Cosinor analysis represents significant variation in the cells transfected with NC inhibitor (P=0.0052) but not miR-96-5p inhibitor (P=0.081). Statistically significant treatment effect was indicated with an asterisk (Student's t-test; *P<0.05). The number of individual data points is the same as the sample size although some points overlap. (d) Endogenous expressions of EAAC1 and β-Actin in HEK293 cells transfected with each miRNA inhibitor are shown. Cells were harvested either 0h or 18h after serum shock. Molecular weight markers are depicted to the right; (e) Quantification of the data in (d) by densitometry is shown. Data represent mean values ± s.e.m obtained from six independent experiments and were analyzed by Wilcoxon rank-sum test. Statistical difference relative to NC inhibitor is indicated with an asterisk (*P<0.05). (f) Effects of miRNA inhibitors on GSH level in HEK293 cells are shown (n = 8, each condition). Cells were harvested either 0h or 18h after serum shock. Data represent mean values ± s.e.m and were analyzed by Wilcoxon rank-sum test. Statistical difference relative to NC inhibitor is indicated with an asterisk (*P<0.05).

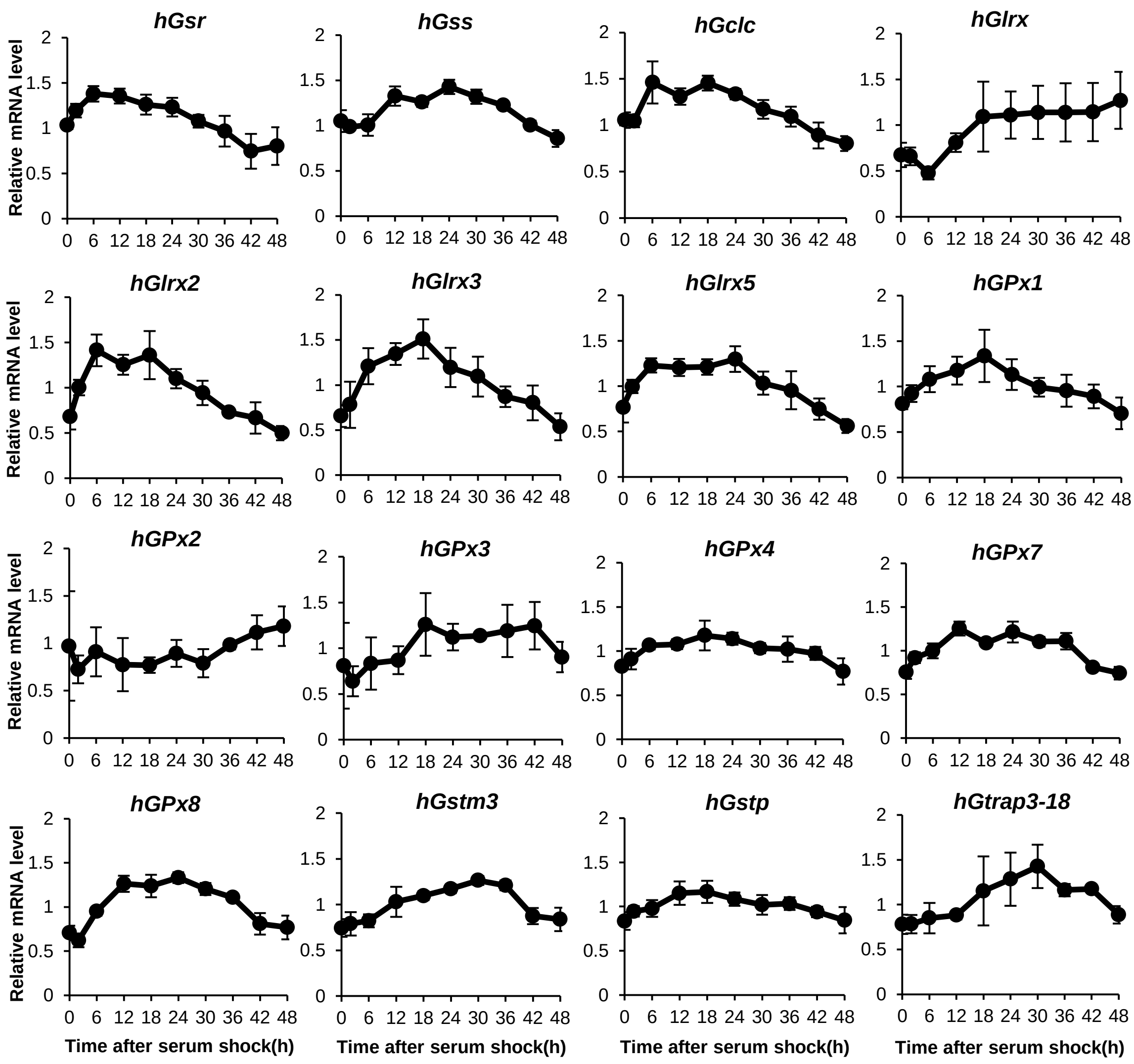

Supplementary Figure 5

Expression patterns of genes involved GSH regulation in serum-shocked SH-SY5Y cells

The result of qRT-PCR for genes involved GSH regulation in SH-SY5Y cells are shown (n=4, each points). Data represent mean values ± s.e.m and were analyzed by a one-way ANOVA and cosinor analysis. No GSH regulation genes show significant circadian rhythm. P-values of each mRNA expression pattern are noted in Supplementary Table 1. We also tried to measure mRNA expressions of *GPx5* and *GPx6* but could not be detected.

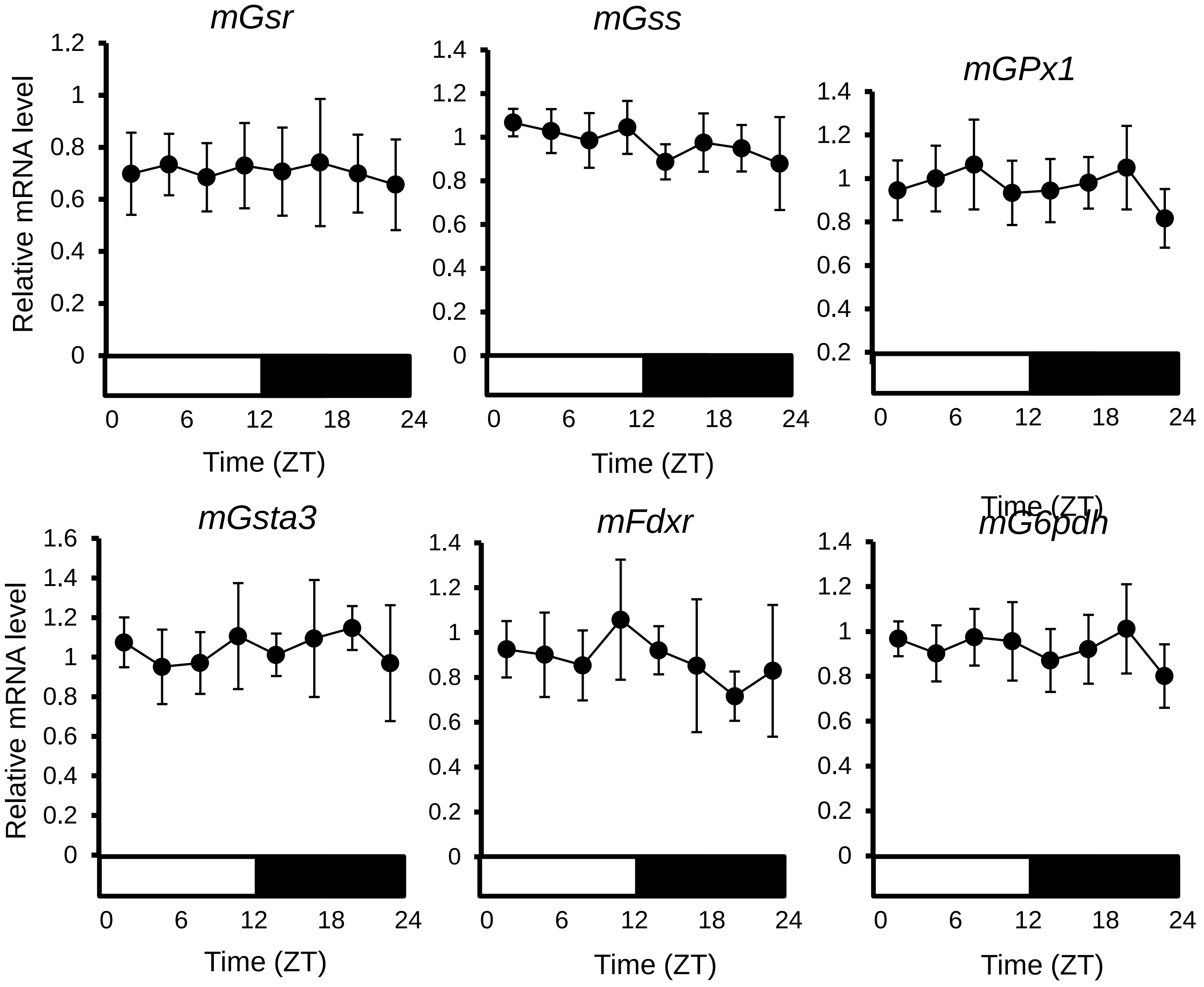

Supplementary Figure 6

Expression patterns of genes involved GSH regulation in SNc

Expression profiles of Glutatione Reductase *(Gsr)*, Glutathione Synthase *(Gss)*, Glutathione Peroxdidase 1 *(Gpx1)*, Glutathione-S-Transferase a3 *(Gsta3)*, Glucose-6-phosphate dehydrogenase *(G6pdh)* and Ferredoxin *(Fdxr)* mRNAs normalized by *Gapdh* expression over 24 h in mesencephalon examined by qRT-PCR (n=8, each point). The bar below the graph indicates the light (white) and dark (black) periods. Data represent mean values ± s.e.m and were analyzed by a one-way ANOVA and cosinor analysis. No GSH regulation genes show significant circadian rhythm. P-values of each mRNA expression pattern are noted in Supplementary Table 1.

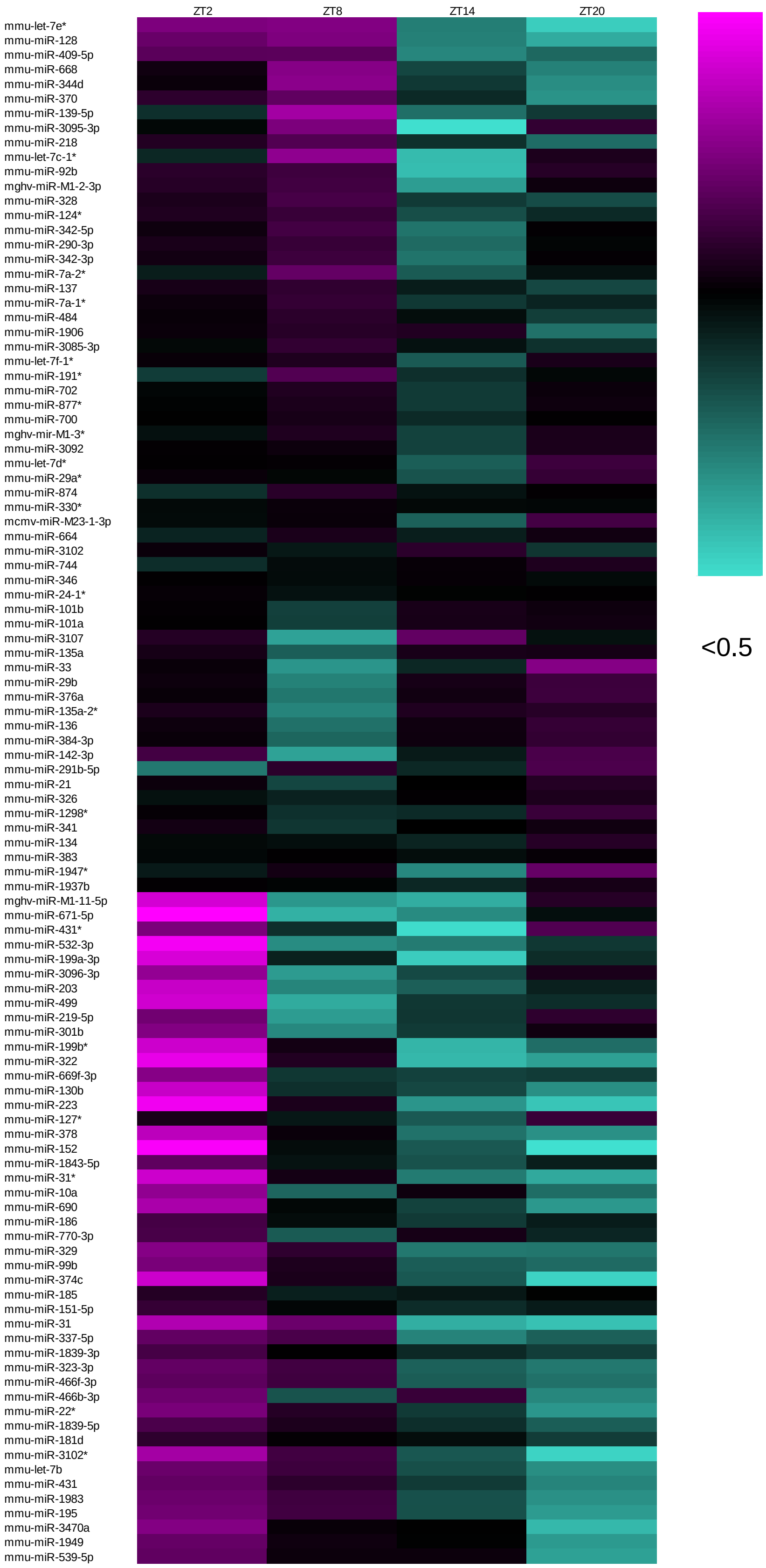

Supplementary Figure 7

Heat map of rhythmic miRNA expression profile with low amplitude in SNc

Patterns of miRNA expression over 24 h in mesencephalon were analyzed using miRNA microarray. Colorgram for the relative levels of miRNAs are shown. The heat scale at the right of the panel represents on a linear scale, where magenta, black and turquoise represent high, average and low expression, respectively.

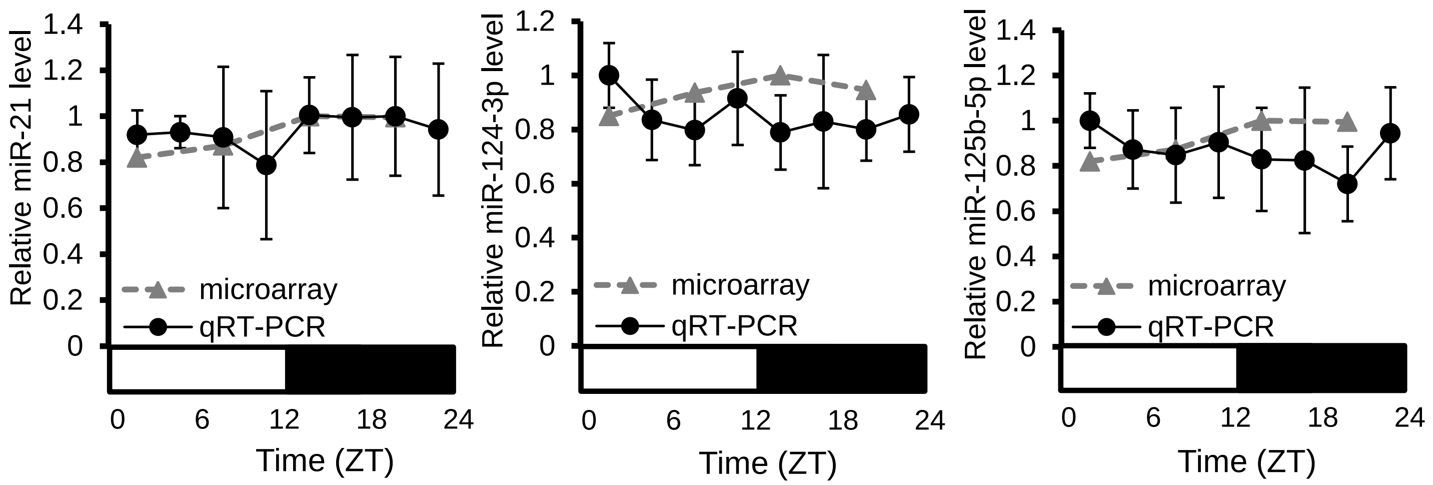

Supplementary Figure 8

Profiles of miRNA expression with no diurnal rhythm

Expression profiles of miR-21, miR-124-3p and miR-125b-5p over 24 h in mesencephalon examined by microarray or qRT-PCR are shown. Data represent mean values ± s.e.m obtained from eight independent experiments and were analyzed by a one-way ANOVA and cosinor analysis. No miRNAs show significant circadian rhythm. P-values of each mRNA expression pattern are noted in Supplementary Table S1.

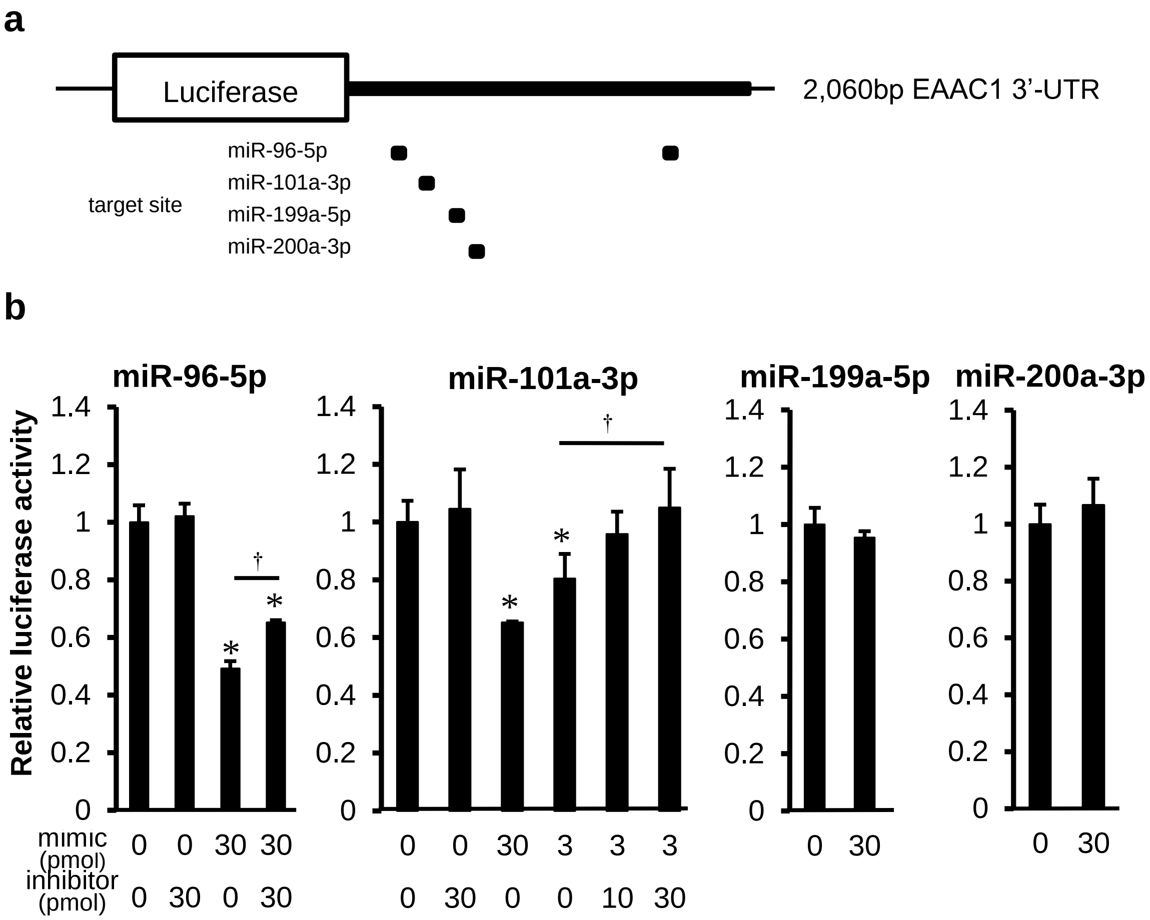

Supplementary Figure 9

Effect of miRNA transfection on luciferase reporter gene assay using mouse EAAC1 3'-UTR

(a) A schematic plot of the luciferase constructs of mouse EAAC1 3'-UTR. The sequences for the target regions of miR-96-5p, miR-101a-3p, miR-199a-5p and miR-200a-3p on EAAC1 3'-UTR are shown. (b) Effects of transfection of each miRNA candidates on luciferase activity using luciferase constructs in (a) are shown. Data represent mean values ± s.e.m and were analyzed by Williams' test. Statistical difference relative to negative control was indicated with asterisk. (**P<0.025*) Dagger mark indicates significant effect of miRNA inhibitor.(†*P<0.025*)

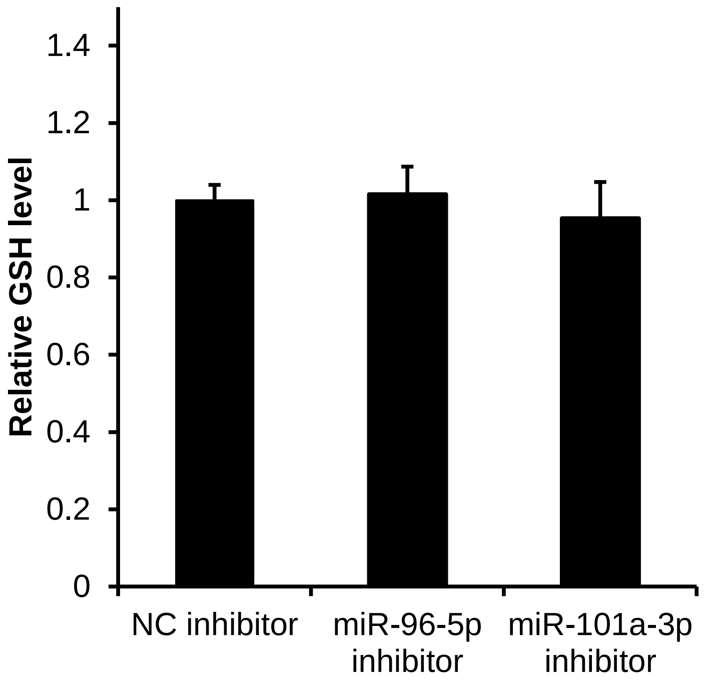

Supplementary Figure 10

Transfection of miRNA inhibitor into SH-SY5Y cells

Effects of miR-96-5p and miR-101a-3p on the GSH level in SH-SY5Y cells (n=3 for each condition) are shown. Data are mean values ± s.e.m. No significant differences were found.

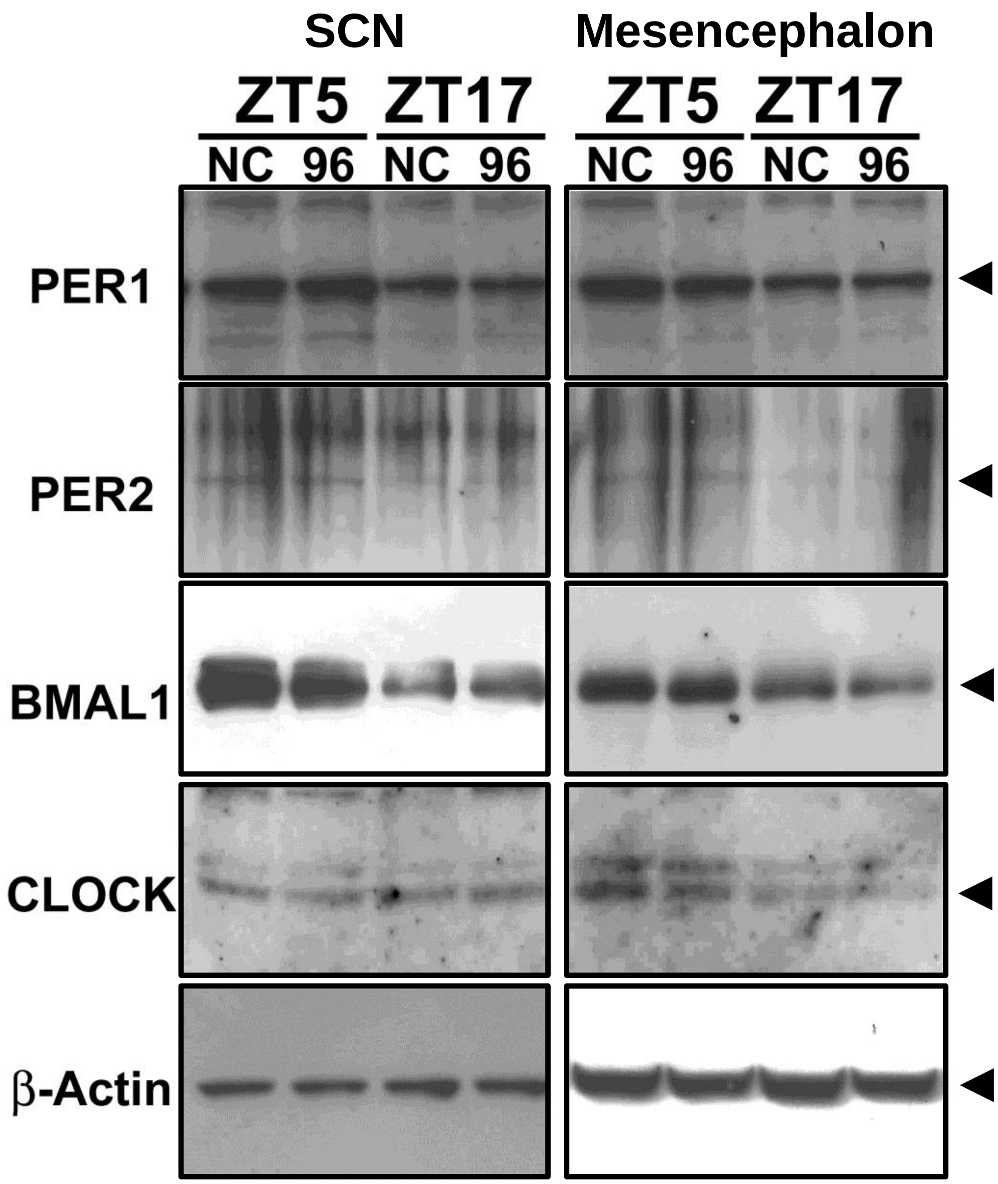

Supplementary Figure 11

Effects of miR-96-5p inhibitor injections on the expression of core clock components

Immunoblots of PER1, PER2, BMAL1, CLOCK and β-Actin in SCN or mesencephalon are shown. Brain tissues were collected at ZT5 or ZT17 a week after intracerebroventricular injection of either negative control (NC) inhibitor or miR-96-5p inhibitor (96) (n=4, each condition). No obvious change in clock protein expression between negative control and miR-96-5p inhibitor injection. Arrows represent approximate molecular size of each clock component estimated by molecular weight markers.

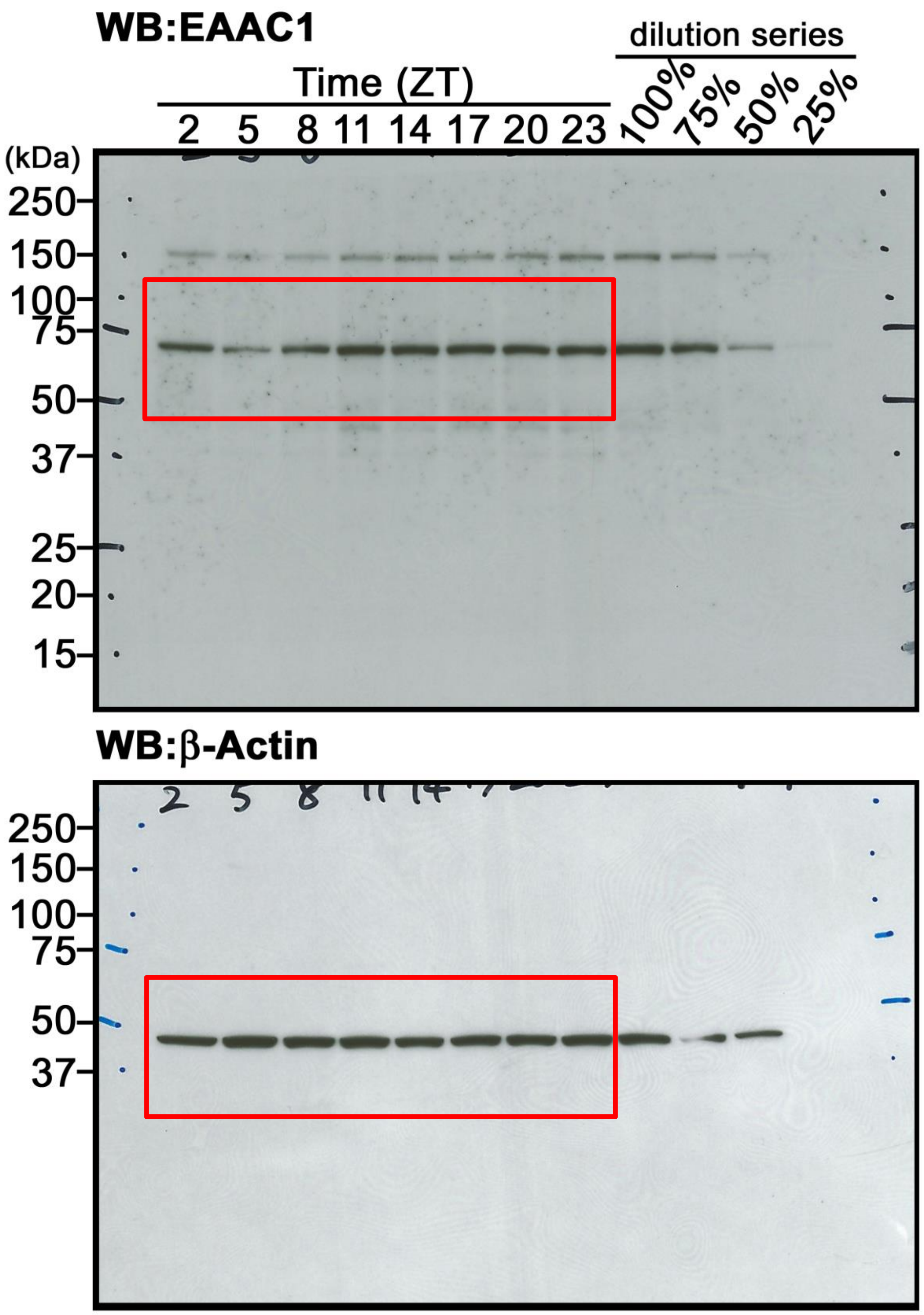

Supplementary Figure 12

Original blots presented in Fig. 2b

Full-length blots of mesencephalic samples at ZT2-23 and the dilution series (100%, 75%, 50% and 25% of ZT14 sample) are shown. Molecular weight markers are indicated on the left. Red squares indicate cropped sections.

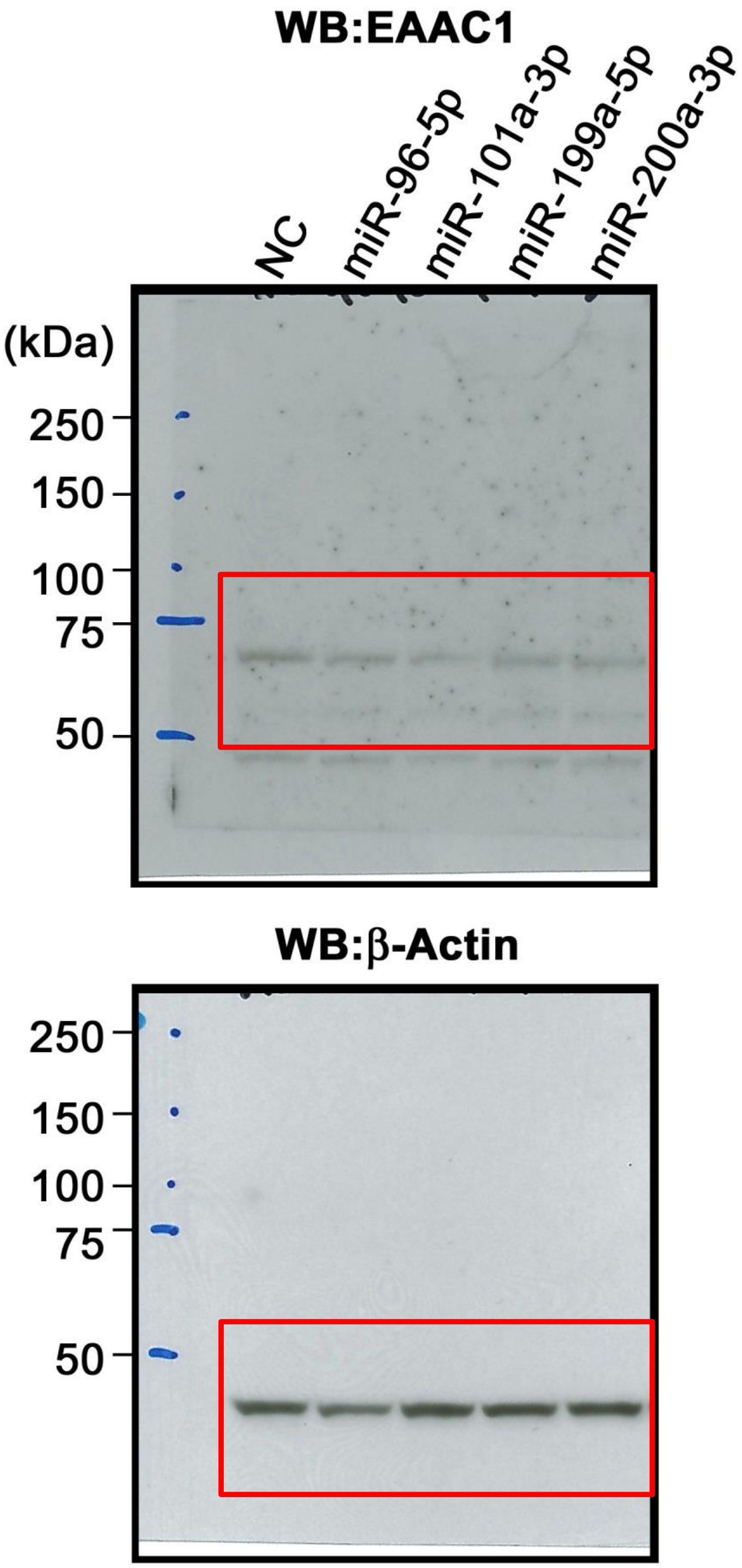

Supplementary Figure 13

Original blots presented in Fig. 4a

Full-length blots of each miRNA effect on EAAC1 expression are shown. Molecular weight markers are indicated on the left. Red squares indicate cropped sections.

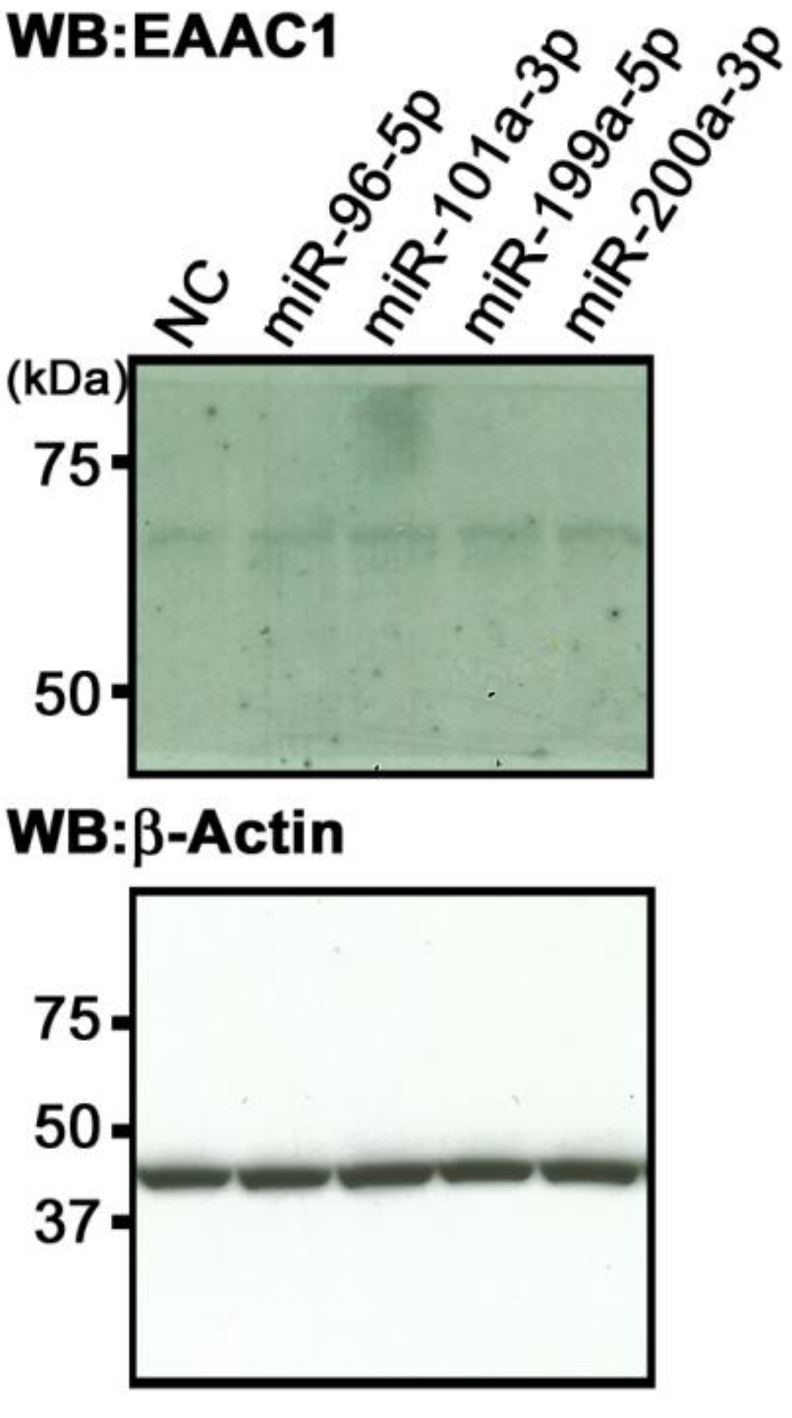

Supplementary Figure 14

Blocking the negative effect of miRNA mimic by miRNA inhibitor on EAAC1 expression

Endogenous expressions of EAAC1 and β-Actin in HEK293 cells transfected with each miRNA mimic and inhibitor are shown. Molecular weight markers are depicted to the right. Quantitation of this data by densitometry is shown in Fig. 4b.

Supplementary Table 1

Rhythmicity in GSH level

Figure	Data	tissue or cell	ANOVA	Cosinor	n
1a	GSH	Mesencephalon	0.000924	0.000182	10
1b	GSSG	Mesencephalon	0.027358	0.002138	8
1c	GSSG/GSH	Mesencephalon	0.461437	0.571775	8
1d	GSH	SH-SY5Y	<0.000001	0.000226	10
Suppl. 1	GSH	SCN	0.340886	0.037003	8
Suppl. 4b	GSH	HEK293 (NC inhibitor)	0.000012	0.001137	8
Suppl. 4b	GSH	HEK293 (miR-96-5pinhibitor)	0.007716	0.055054	8

Rhythmicity in Cell Viability

Figure	Data	tissue or cell	ANOVA	Cosinor	n
1e	Cell Viability	SH-SY5Y	<0.000001	0.000297	7
Suppl. 4c	Cell Viability	HEK293 (NC inhibitor)	0.002384	0.005297	4
Suppl. 4c	Cell Viability	HEK293 (miR-96-5p inhibitor)	0.115258	0.081402	4

Rhythmicity in mRNA level

Figure	mRNA	Tissue or Cell	ANOVA	Cosinor	n
Suppl.2a	*Per2*	Mesencephalon	0.000003	0.000605	8
Suppl. 6	*Gsr*	Mesencephalon	0.874201	0.725501	8
Suppl. 6	*Gss*	Mesencephalon	0.052356	0.070672	8
Suppl. 6	*GPx1*	Mesencephalon	0.122992	0.529546	8
Suppl. 6	*Gsta3*	Mesencephalon	0.357222	0.039644	8
Suppl. 6	*Fdxr*	Mesencephalon	0.174972	0.089987	8
Suppl. 6	*G6pdh*	Mesencephalon	0.195117	0.776112	8
Suppl. 2b	*Per2*	SH-SY5Y	<0.000001	0.002812	4
Suppl. 5	*Gsr*	SH-SY5Y	0.000017	0.997273	4
Suppl. 5	*Gss*	SH-SY5Y	<0.000001	0.627173	4
Suppl. 5	*Gclc*	SH-SY5Y	0.000006	0.300709	4
Suppl. 5	*Glrx*	SH-SY5Y	0.000579	0.569456	4
Suppl. 5	*Glrx2*	SH-SY5Y	<0.000001	0.280390	4
Suppl. 5	*Glrx3*	SH-SY5Y	0.000003	0.957848	4
Suppl. 5	*Glrx5*	SH-SY5Y	<0.000001	0.086899	4
Suppl. 5	*GPx1*	SH-SY5Y	0.001835	0.114141	4
Suppl. 5	*GPx2*	SH-SY5Y	0.243749	0.659397	4
Suppl. 5	*GPx3*	SH-SY5Y	0.061544	0.943910	4
Suppl. 5	*GPx4*	SH-SY5Y	0.000416	0.068056	4
Suppl. 5	*GPx7*	SH-SY5Y	<0.000001	0.065837	4

(continued)

Figure	mRNA	Tissue or Cell	ANOVA	Cosinor	n
Suppl. 5	*GPx8*	SH-SY5Y	<0.000001	0.106429	4
Suppl. 5	*Gstm3*	SH-SY5Y	<0.000001	0.208546	4
Suppl. 5	*Gstp*	SH-SY5Y	0.002996	0.658957	4
Suppl. 5	*Gtrap3-18*	SH-SY5Y	0.770391	0.594399	4

Rhythmicity in miRNA level

Figure	miRNA	Tissue or Cell	ANOVA	Cosinor	n
3b	miR-96-5p	Mesencephalon	0.0062884	0.008218	8
3c	miR-101a-3p	Mesencephalon	0.0323427	0.052662	8
3d	miR-199a-5p	Mesencephalon	0.0198431	0.025831	8
3e	miR-200a-3p	Mesencephalon	0.0021950	0.023342	8
Suppl. 8	miR-21	Mesencephalon	0.7876359	0.354657	8
Suppl. 8	miR-124-3p	Mesencephalon	0.4463925	0.330269	8
Suppl. 8	miR-125b-5p	Mesencephalon	0.1932641	0.138306	8

P value from ANOVA or Cosinor fitting test

ANOVA; p-value from one-way ANOVA

Cosinor; p-value from cosinor analysis

Supplementary Table 2

Figure	Gene	Tissue or Cell	Primer Sequence
2a	*Eaac1*	Mesencephalon	Fw: 5'-TCACCACAGCCATGACAACA-3'<br>Rv: 5'-CCCCTTTTCTCCCATTTTCC-3'
Suppl. 2a	*Per2*	Mesencephalon	Fw: 5'-CTCAGGAGTGCATGGAGGAG-3'<br>Rv: 5'-TTGGTGTGTGGGTTGTTGTG-3'
Suppl. 6	*Gsr*	Mesencephalon	Fw: 5'-GTGCCAACAAAGAGGAAAAGG-3'<br>Rv: 5'-CCCCATTTTCACCGCTACA-3'
Suppl. 6	*Gss*	Mesencephalon	Fw: 5'-CAGCTCGAAGAACTGGCAAAG-3'<br>Rv: 5'-AGGGGAAAAGCGTGAATGG-3'
Suppl. 6	*GPx1*	Mesencephalon	Fw: 5'-ACTGGTGGTGCTCGGTTTC-3'<br>Rv: 5'-CACCATTCACTTCGCACTTCTC-3'
Suppl. 6	*Gsta3*	Mesencephalon	Fw: 5'-GGCAAGGTTACGAAGTGATGG-3'<br>Rv: 5'-CAATGTAGTTGAGAATGGCTTTGG-3'
Suppl. 6	*Fdxr*	Mesencephalon	Fw: 5'-GAAGCAGCTCGTGCCATTC-3'<br>Rv: 5'-GGGCTGTCTGTGTAAATGTGTTG-3'
Suppl. 6	*G6pdh*	Mesencephalon	Fw: 5'-GCCACCCATCTTTCCACAA-3'<br>Rv: 5'-TCCTCCCCATTGTTCCTAACTC-3'
Suppl. 6	*Gapdh*	Mesencephalon	Fw: 5'-AAAATGGTGAAGGTCGGTGTG-3'<br>Rv: 5'-AATGAAGGGGTCGTTGATGG-3'
Suppl. 2b	*Per2*	SH-SY5Y	Fw: 5'-TTATGAGCCAGACCTCCTCCA-3'<br>Rv: 5'-CTGATGACGCTGTCCAAGCA-3'
Suppl. 5	*Gsr*	SH-SY5Y	Fw: 5'-GTATCACGCAGTTACCAAAAGGAA-3'<br>Rv: 5'-CAGCATTTCATCACACCCAAG-3'
Suppl. 5	*Gss*	SH-SY5Y	Fw: 5'-CAGTGTCCTGAGTAAGACCAAAGAA-3'<br>Rv: 5'-GCAATCAGTAGCACCAGAGCA-3'
Suppl. 5	*Gclc*	SH-SY5Y	Fw: 5'-AAAACATGGAAGTGGATGTGGA-3'<br>Rv: 5'-TTGCTTGTAGTCAGGATGGTTTG-3'
Suppl. 5	*Glrx*	SH-SY5Y	Fw: 5'-ACAAGGGCTTCTGGAATTTGTC-3'<br>Rv: 5'-GCATCCGCCTATACAATCTTTACC-3'
Suppl. 5	*Glrx2*	SH-SY5Y	Fw: 5'-CCTGTGAACCAGATCCAAGAAAC-3'<br>Rv: 5'-AGCAGGTCCAGTTCCACCA-3'
Suppl. 5	*Glrx3*	SH-SY5Y	Fw: 5'-ACTCAAATTGGCCAACATACCC-3'<br>Rv: 5'-CACCATTTTCTTTCAGTTCCTTCAC-3'
Suppl. 5	*Glrx5*	SH-SY5Y	Fw: 5'-CCTACAACGTGCTGGACGAC-3'<br>Rv: 5'-TTCCACCAAGTCCCCATTC-3'

(continued)

Figure	Gene	Tissue or Cell	Primer Sequence	
Suppl. 5	*GPx1*	SH-SY5Y	Fw:<br>Rv:	5'-AGAATGTGGCGTCCCTCTG-3'<br>5'-AATCTCTTCGTTCTTGGCGTTC-3'
Suppl. 5	*GPx2*	SH-SY5Y	Fw:<br>Rv:	5'-TCATCATTTGGAGCCCTGTG-3'<br>5'-ATGTTGATGGTTGGGAAGGTG-3'
Suppl. 5	*GPx3*	SH-SY5Y	Fw:<br>Rv:	5'-AGTATGCTGGCAAATACGTCCTC-3'<br>5'-AAGCCCAGAATGACCAGACC-3'
Suppl. 5	*GPx4*	SH-SY5Y	Fw:<br>Rv:	5'-CGCTGTGGAAGTGGATGAAG-3'<br>5'-CTTGTCGATGAGGAACTTGGTG-3'
Suppl. 5	*GPx5*	SH-SY5Y	Fw:<br>Rv:	5'-TCCATCTGCTTCCCCTTCTC-3'<br>5'-GGTGCCTTTCTCGTCTTTGTG-3'
Suppl. 5	*GPx6*	SH-SY5Y	Fw:<br>Rv:	5'-TGGAAAACAAGAACCAGGAACA-3'<br>5'-GCTGGAAACTGGGGACAAAG-3'
Suppl. 5	*GPx7*	SH-SY5Y	Fw:<br>Rv:	5'-AAACTGGTGTCGCTGGAGAAG-3'<br>5'-TCGGTAGTGCTGGTCTGTGAA-3'
Suppl. 5	*GPx8*	SH-SY5Y	Fw:<br>Rv:	5'-GGAGAACCTGCATTTAGATTTCTTG-3'<br>5'-AATGGGCTCCTCTGGCTTC-3'
Suppl. 5	*Gstm3*	SH-SY5Y	Fw:<br>Rv:	5'-CAGAACCGTATATTTGACCCCAAG-3'<br>5'-GGCAGCGATTTTCTCCAAAG-3'
Suppl. 5	*Gstp*	SH-SY5Y	Fw:<br>Rv:	5'-AACTATGAGGCGGGCAAGG-3'<br>5'-CAGCAGGTTGTAGTCAGCGAAG-3'
Suppl. 5	*Gtrap3-18*	SH-SY5Y	Fw:<br>Rv:	5'-AACCGCGTAGTGAGCAACC-3'<br>5'-AACCCTGTGAACACCAGCAC-3'
Suppl. 5	*Gapdh*	SH-SY5Y	Fw:<br>Rv:	5'-GAAGGTGAAGGTCGGAGT-3'<br>5'-GAAGATGGTGATGGGATTTC-3'

List of primers used for quantitative RT-PCR

Fw: Forward primer

Rv: Reverse primer
